# Deposition of uniform films on complex 3D objects by atomic layer deposition for plasma etch-resistant coatings

**DOI:** 10.1093/nsr/nwaf247

**Published:** 2025-06-17

**Authors:** Xin Han, Yixian Wang, Yumo Tian, Yafeng Wang, Lipei Peng, Chunlei Pei, Tuo Wang, Jinlong Gong

**Affiliations:** School of Chemical Engineering & Technology, Key Laboratory for Green Chemical Technology of Ministry of Education, Tianjin University; Collaborative Innovation Center for Chemical Science & Engineering; Tianjin 300072, China; International Joint Laboratory of Low-carbon Chemical Engineering of Ministry of Education, Tianjin 300350, China; School of Chemical Engineering & Technology, Key Laboratory for Green Chemical Technology of Ministry of Education, Tianjin University; Collaborative Innovation Center for Chemical Science & Engineering; Tianjin 300072, China; International Joint Laboratory of Low-carbon Chemical Engineering of Ministry of Education, Tianjin 300350, China; School of Chemical Engineering & Technology, Key Laboratory for Green Chemical Technology of Ministry of Education, Tianjin University; Collaborative Innovation Center for Chemical Science & Engineering; Tianjin 300072, China; International Joint Laboratory of Low-carbon Chemical Engineering of Ministry of Education, Tianjin 300350, China; School of Chemical Engineering & Technology, Key Laboratory for Green Chemical Technology of Ministry of Education, Tianjin University; Collaborative Innovation Center for Chemical Science & Engineering; Tianjin 300072, China; Peric Special Gases Co., Ltd, Handan 056002, China; Peric Special Gases Co., Ltd, Handan 056002, China; School of Chemical Engineering & Technology, Key Laboratory for Green Chemical Technology of Ministry of Education, Tianjin University; Collaborative Innovation Center for Chemical Science & Engineering; Tianjin 300072, China; International Joint Laboratory of Low-carbon Chemical Engineering of Ministry of Education, Tianjin 300350, China; National Industry-Education Platform of Energy Storage, Tianjin University, Tianjin 300350, China; Haihe Laboratory of Sustainable Chemical Transformations, Tianjin 300192, China; Joint School of National University of Singapore and Tianjin University, International Campus of Tianjin University, Fuzhou 350207, China; Zhejiang Institute of Tianjin University, Ningbo, 315201, China; State Key Laboratory of Synthetic Biology, Tianjin University, Tianjin 300072, China; School of Chemical Engineering & Technology, Key Laboratory for Green Chemical Technology of Ministry of Education, Tianjin University; Collaborative Innovation Center for Chemical Science & Engineering; Tianjin 300072, China; International Joint Laboratory of Low-carbon Chemical Engineering of Ministry of Education, Tianjin 300350, China; National Industry-Education Platform of Energy Storage, Tianjin University, Tianjin 300350, China; Haihe Laboratory of Sustainable Chemical Transformations, Tianjin 300192, China; Joint School of National University of Singapore and Tianjin University, International Campus of Tianjin University, Fuzhou 350207, China; Zhejiang Institute of Tianjin University, Ningbo, 315201, China; State Key Laboratory of Synthetic Biology, Tianjin University, Tianjin 300072, China; School of Chemical Engineering & Technology, Key Laboratory for Green Chemical Technology of Ministry of Education, Tianjin University; Collaborative Innovation Center for Chemical Science & Engineering; Tianjin 300072, China; International Joint Laboratory of Low-carbon Chemical Engineering of Ministry of Education, Tianjin 300350, China; State Key Laboratory of Synthetic Biology, Tianjin University, Tianjin 300072, China; Tianjin Normal University, Tianjin 300387, China

**Keywords:** atomic layer deposition, thin film, film uniformity, 3D object deposition, plasma etching, etch-resistant coating

## Abstract

Atomic layer deposition (ALD) is a layer-by-layer technique for producing conformal, high-quality films, ideal for corrosion-resistant coatings on complex geometries. However, depositing thicker films on complex 3D objects presents challenges in maintaining effective precursor delivery across the surface. This paper describes the design and realization of a method to increase precursor concentration in the boundary layer near the complex object surface by introducing slitted baffles within the chamber. Using –OH surface coverage as a key indicator for assessing the extent of surface reactions, simulation is used to optimize the baffle number and shape. The optimal baffle design reduced surface film non-uniformity on the object surface from 35.46% to 5.75%, shortened the purge time from 12.8 to 9.7 s, and increased precursor utilization by 7%. Ideal Al_2_O_3_ films exhibited a fluorinated plasma etching rate of 1.11 nm min^−1^, five times stronger than non-ideal films (5.19 nm min^−1^), indicating superior plasma etching resistance.

## Teaser

This paper proposes a method to increase precursor concentration near complex surfaces and enhance mass transfer by adding slitted baffles in the ALD chamber.

## INTRODUCTION

As integrated circuit technology has advanced, the reduction in the half spacing of semiconductor devices has increased the importance of controlling contamination by particulate pollutants [1]. Fluorine-containing plasmas, commonly used in semiconductor processing, can erode metallic parts within plasma etching chambers, generating particles that not only degrade equipment performance but also contaminate wafers [2–4]. Consequently, extensive research has been focused on the development of plasma-resistant materials, such as Y_2_O_3_ and Al_2_O_3_, along with the advancement of coating technologies [2]. Currently, plasma-resistant coatings are primarily produced through vacuum kinetic spray [5] or thermal spraying methods, such as atmospheric plasma spraying (APS) [6] and suspension plasma spray (SPS) [7]. However, thermal spraying is limited by its inability to coat complex structures, leaving critical components, such as nozzles, unprotected. Atomic layer deposition (ALD) offers an alternative solution. As a specialized chemical vapor deposition (CVD) technique, ALD is featured by a self-limiting growth mechanism, enabling conformal and step coverage on workpieces with high aspect ratio structures [8–12]. This makes ALD an ideal method to coat protective layers for plasma etching resistance. ALD coatings have already proven effective in applications requiring corrosion resistance, atomic oxygen resistance in aerospace, and plasma etching resistance [1,13–16]. However, as the complexity of the surface geometry increases, achieving uniform coating thickness becomes more challenging, highlighting the need for precise control in ALD processes [17,18].

ALD allows for precise control of film thickness at the atomic level [19–22]. However, as the number of deposition cycles increases, maintaining film uniformity becomes increasingly difficult across the complex objects [23,24], primarily because of the variation of the flow field distribution within the reactor under different temperatures and pressures [25–27]. Researchers have achieved uniform velocity, temperature and concentration fields by adding internal components or altering the reactor design, enabling the prediction of uniform film deposition and facilitating the scaling up of ALD deposition for 2D planar surfaces [28–30]. For example, Christofides *et al.* designed an optimized reactor configuration by modifying the inlet geometry to ensure ideal fluid dynamic conditions, such as minimal vortex formation and uniform radial flow distribution. This approach effectively addressed the issue of uneven film distribution caused by spatial steric hindrance for 250-mm diameter wafer samples [31]. Our previous work has improved fluid velocity and temperature distribution by adding a circular gas distributor and optimizing its size and placement, achieving uniform deposition across large substrate areas [non-uniformity (NU) < 0.88%] [32]. These studies have primarily concentrated on scaling up of ALD deposition on 2D surfaces [29,30,33]. However, maintaining uniform deposition becomes considerably more difficult on 3D parts with complex geometries and high curvature, which are widely used in semiconductor-manufacturing equipment [34].

Achieving uniform thin-film deposition on large-area, high-curvature surfaces requires not only uniform flow fields within the reactor but also an adequate supply of precursors. Fryauf and colleagues focused on the design of substrates and chamber walls for large-scale ALD systems, such as those used for curved substrates like telescope mirrors. They demonstrated that optimizing flow-diverting sheet geometries could reduce flow pattern inconsistencies and improve deposition uniformity within the chamber [35]. However, achieving uniform deposition on complex 3D objects with large surface curvature, such as showerheads, remains challenging [36–38]. To address this challenge, designing reaction chambers with tailored geometries is essential. For example, Liu *et al.* developed a self-built reactor capable of double-sided coating on large, highly curved glass substrates. They identified the key to improving film uniformity as ensuring consistent gas flow across the surface [39]. However, even with uniform flow fields, variations in precursor adsorption probabilities across workpieces can still arise. To overcome this, an adequate supply of precursors and thorough purging are essential to prevent simultaneous presence of the two precursors in the chamber, which could otherwise reduce precursor utilization efficiency.

Process conditions such as temperature, pressure, precursor pulse time and purge time are critical for maximizing precursor utilization during ALD thin-film deposition [34,40]. To achieve ideal ALD growth, it is crucial to provide both an adequate precursor supply and sufficient purging time; otherwise, non-ideal growth modes, such as CVD-like growth, may occur, compromising film quality [41–43]. When an ALD coating is applied as a protective layer, any reduction in film quality can significantly compromise its protective performance. To address this, researchers have investigated the effects of temperature and pressure on the reaction, optimized precursor dosing times to ensure sufficient precursor supply with minimal waste, and determined the minimum purge time required to improve process efficiency [44–47]. Chen and co-workers employed a combination of surface chemistry and quantitative fluid dynamics models to explore the influence of temperature, precursor mass fraction, mass flow rate, and pressure on the optimization of the transient process. Their findings highlight the critical roles of precursor concentration and gas velocity in process optimization. They also indicate that precursors in the boundary layer significantly contribute to surface reactions [48]. Furthermore, their findings highlight that precursor contributions to surface reactions primarily occur within the boundary layer, where the precursor concentration is lower than in the bulk chamber. This indicates that most precursors are swept out of the chamber with the bulk gas flow. For deposition on large, complex 3D surfaces, precursor utilization remains low, even under minimal dose times, presenting a significant challenge for achieving efficient and uniform deposition.

This paper describes the design and realization of a type of simple yet highly effective slitted baffle for ALD reactors to control the local precursor concentration in the surface boundary layer. This advancement realized the uniform thin-film deposition on complex 3D objects, such as showerhead-shaped structures, while improving precursor utilization. In contrast to traditional methods that mainly focused on flow field predictions to assess film deposition uniformity, this study employs –OH surface coverage as a more representative indicator for uniformity evaluation. Without altering the precursor input amount, the clearance of the slit between the baffle and the object was found to impose significant impacts on film quality and uniformity. An optimized slit clearance increased the precursor concentration within the fluid boundary layer on the sample surface while maintaining a small pressure drop throughout the reactor, avoiding non-ideal ALD processes. As a result, precursor utilization decreased by approximately 5% and the precursor purge time decreased from 12.8 to 9.7 s, while the predicted film NU over the 3D parts based on the –OH surface coverage was improved from 11.73% to 1.63%, which correlated with an experimentally measured film thickness NU reduction from 35.46% to 5.75%. Meanwhile, upon the addition of baffles, the plasma etching rate of the Al_2_O_3_ film improved from 5.19 nm min^−1^ (non-ideal ALD) to 1.1 nm min^−1^, showing the importance of achieving ideal ALD deposition over the entire 3D object to enhance its plasma etching resistance.

## RESULTS AND DISCUSSION

**Figure 1.**
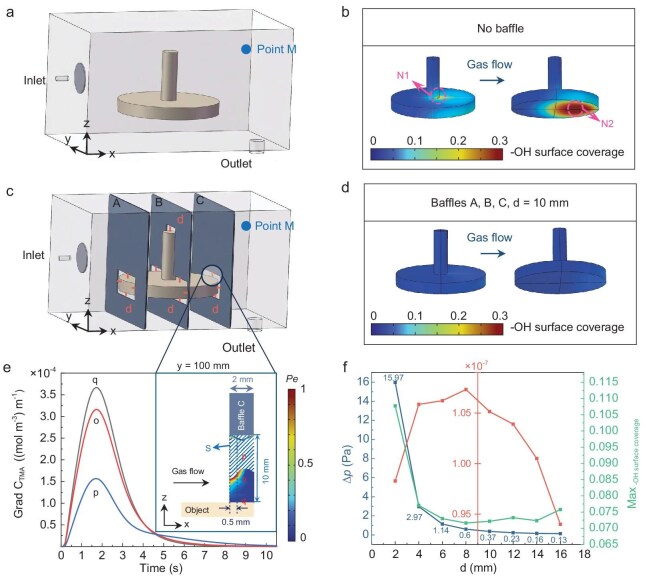
Effect of baffle addition on surface reactions. (a) Position of the showerhead-shaped sample in the chamber. (b) –OH surface coverage on the sample when no baffles are added. (c) Relative position of the Baffles A, B and C to the sample. (d) –OH surface coverage on the sample after adding Baffles A, B and C. (e) Concentration gradients at p, o and q and the region within the slit (Region S) along the *z*-axis where *Pe* < 1. (f) The *∆p* within the chamber, the maximum surface –OH coverage and the TMA concentration at purge point M after the first half-reaction when adding Baffles A, B and C.

In the deposition of complex 3D objects within a chamber, the probability of precursor molecules reaching various surface areas varies, often resulting in uneven film formation. To address this, flow and temperature distributions are simulated under steady-state conditions, while precursor transport is modeled under transient conditions to accurately describe the time-dependent behavior of precursor delivery and surface reaction (details in the online supplementary file, Section S1.1). The boundary conditions of simulation include an inlet flow rate of 400 sccm and an outlet pressure of 170 Pa, while a circular baffle is added at the inlet to promote a uniform flow field throughout the chamber [32]. Simulations incorporate a complex 3D sample, resembling a showerhead without holes, centrally positioned within the chamber (Fig. 1a). This configuration generates a stable flow field across the sample surface, free from disruptive eddy currents (Fig. S12 in the online supplementary file). For a more detailed analysis of precursor consumption and reaction kinetics during the ALD process, a half-reaction model is used to predict the depletion of the trimethylaluminum (TMA) precursor and the extent of the surface reaction. During the first half-reaction of Al_2_O_3_ deposition, surface hydroxyl groups (–OH) are progressively consumed, and if all surface –OH is consumed during this half-reaction, the –OH surface coverage gradually decreases from unity (1) at the start of the reaction to 0 by the end of the first half-reaction (TMA dose). The purge effectiveness is evaluated using the purge time at point M, located 20 mm (10% of the chamber height) from the top, above the outlet (Fig. 1a). Adequate purging is defined as the moment when the TMA concentration at point M falls below 1 × 10^−^^7^ mol m^−3^. After placing the complex object (a sample made of a Φ165/20 mm disk with a Φ25/80 mm rod) in the chamber with a 0.0075 mol m^−3^ TMA pulse for 0.02 s without any additional internal components, simulation shows that when the TMA is fully purged from the chamber, the surface –OH coverage does not reach zero in all areas. In fact, surface –OH near the outlet is not fully consumed (denoted as Region N), with a maximum value of 0.35 (>0, Region N2 in Fig. 1b). These results indicate that the amount of precursor reacting on the sample surface is insufficient without adding flow-regulating baffles when TMA dosing time is 0.02 s. Most of the precursor does not react with the surface and is instead purged out of the chamber, resulting in precursor waste and reduced deposition uniformity.

To enhance precursor utilization and reduce deposition costs, slitted baffles with polytetrafluoroethylene (PTFE) surface mounting were added to increase the amount of precursor reaching the sample surface. Al_2_O_3_ was deposited for 1000 cycles on both PTFE and silicon substrates (the thickness of the Al_2_O_3_ measured on the silicon substrate was 102 nm). X-ray photoelectron spectroscopy (XPS) results reveal a very low Al 2p signal, accounting for only 0.59 atm%, as compared with the dominant F composition (82.68 atm%) from the PTFE, suggesting negligible Al_2_O_3_ deposition on PTFE. In contrast, the 102 nm Al_2_O_3_ on silicon substrate presents a much stronger Al 2p XPS peak, suggesting an Al composition of 10.06 atm%, which is common in ALD-grown Al_2_O_3_ [49], for which the sum of Al and O is less than 100% due to surface contamination after air exposure (Table 1, Fig. S18). These findings demonstrate that the baffle with PTFE does not adsorb precursors, or adsorbs only a negligible amount compared to the sample. In the simulation, it is assumed that the precursor flux at the inlet remains constant, with or without the addition of baffles, and precursor adsorption on the baffle is considered zero. Additionally, the baffle thickness is consistently set at 2 mm in both the simulation and experiment, ensuring that the addition of baffles will not induce vortex formation within the chamber, thereby preserving the integrity of the purge process (Fig. S12). Without the addition of baffles, regions where reactions are less likely to occur are found at the Regions N1 and N2 (Fig. 1b), indicating incomplete –OH consumption at these two regions. The highest surface –OH coverage is observed in Region N2, primarily due to the limited precursor availability beneath the sample, as the majority of the precursor is distributed to the upper surface during dosing. To address this issue, Baffle A is introduced to achieve a more uniform distribution of the precursor across both the upper and lower surfaces of the sample (Fig. 1c; Fig. S1). Additionally, Baffles B and C (Baffle C is identical in size to A) are placed near Regions N1 and N2, respectively, locations with high –OH surface coverages where the adsorption of precursors is insufficient for surface reaction (Fig. 1c; Fig. S1). Assuming that Baffles A, B and C are all introduced, the –OH on the surface of the 3D object can be effectively consumed (Fig. 1d). The slit clearance (denoted as d) between the baffle and the sample surface (Fig. 1c; Fig. S18d) influences the surface reaction, and there exists an optimal d that maximizes precursor utilization, which is defined as the ratio of the consumed amount (the total inflow at the inlet minus the total outflow at the outlet) to the total inflow at the inlet.

**Table 1.** Atomic ratio in XPS data.

	Deposition	Al	O	Si	F
Substrate	cycles	(atm%)	(atm%)	(atm%)	(atm%)
Si	1000	10.06	81.18		
PTFE	1000	0.59	5.64		82.68

The optimal slit clearance is determined by evaluating the pressure drop (*∆p*) across the baffles and the TMA concentration inside the chamber. According to the surface kinetics of Al_2_O_3_ deposition (Section S1.2), the adsorption of the precursor during the first half-reaction (TMA dose) on the surface is related to its partial pressure. When Baffles A, B and C are introduced, pressure drops develop across them within the chamber if d is small (Fig. S8b). The pressure is higher between Baffle A and the chamber inlet, while it is lower between Baffle C and the chamber outlet (Fig. S8b). This results in lower adsorption near the low-pressure area, leading to relatively high –OH coverage, whereas the opposite trend is observed in the high-pressure zone (Fig. 1f; Fig. S8d). As d increases from 2 to 8 mm, the *∆p* decreases and approaches equilibrium, resulting in a gradual reduction in –OH coverage (Fig. 1f; Fig. S8c). Once d = 10 mm, the –OH coverage begins to increase, indicating reduced precursor utilization due to low surface precursor partial pressure. This suggests that as the number of deposition cycles increases, differences in surface adsorption may lead to uneven surface deposition. Hence, when d is >8 mm, the *∆p* is expected to impose minimal impact on surface –OH consumption. With *∆p* < 0.5 Pa, this threshold defines the minimum slit clearance requirement; when d = 8 mm, *∆p* = 0.6 Pa (>0.5 Pa) and when d = 10 mm, *∆p* = 0.37 Pa (<0.5 Pa). The precursor is effectively purged from the chamber after 12.8 s at d = 10 mm with Baffles A, B and C. Under the same purge time, as d increases, the TMA concentration at point M rises (Fig. 1f) due to reduced precursor adsorption as the chamber pressure reaches equilibrium (*∆p* < 0.5 Pa). It then decreases as the larger d reduces gas velocity, enhancing diffusion toward the purge point. This confirms that at d = 10 mm, *∆p* across the baffles in the chamber no longer affects the reaction.

To evaluate the indispensability of Baffles A, B and C, the surface reaction behavior is examined under different baffle number configurations. The surface –OH coverage at the end of the first half-reaction varies with the number of baffles introduced for a constant TMA precursor flux (0.0075 mol m^−3^ TMA pulse for 0.02 s in simulation). For a single baffle configuration (either Baffle A, B or C), adjusting the slit clearance d results in a maximum surface –OH coverage greater than 0.1 (>0) (Figs S2–S4). The surface –OH coverage map (Figs S2–S4) shows that a single baffle is insufficient for complete surface reaction, with unconsumed –OH remaining near the outlet. When the number of baffles is increased to two, the –OH surface coverage after the first half-reaction reveals that adding Baffles A and B results in higher –OH coverage near the outlet on both the upper and lower surfaces of the sample (Fig. S5). Similarly, adding Baffles B and C produces high –OH coverage near the outlet on the lower surface (Fig. S6), while adding Baffles A and C leaves a region of high –OH coverage at the Region N1 (Fig. S7, the same position as in Fig. 1b Region N1). These results indicate that two baffles are still insufficient for complete –OH consumption across the surface. Further increasing the baffle count to three, with baffles positioned at the front, middle and rear of the sample (Fig. 1c, Fig. S8), allows the precursor to flow effectively across the surface of the sample, enhancing mass transfer between the precursor and the surface. A comparison of –OH surface coverage demonstrates that the addition of three baffles results in a more complete surface reaction and improved uniformity (Fig. 1d).

It is hypothesized that the slit clearance affects the exposure of TMA on the sample surface by influencing the ratio of boundary layer thickness to the slit clearance. Based on the optimization of *∆p* within the chamber, it was determined that upon the addition of Baffles A, B and C, the pressure drop is 0.34 Pa (<0.5 Pa) at d = 10 mm, which is considered the minimum slit clearance. As fluid flows through the slit, it transitions from an undeveloped region (also called entrance length, denoted as *L_e_*) to a fully developed region, with a boundary layer forming near the surface. *L_e_* is calculated using Equation (1) derived from the Navier–Stokes equations [50,51]:


(1) \begin{eqnarray*}
{L}_e = 0{\mathrm{.104}}{u}_x{\left( {\frac{{\mathrm{d}}}{{\mathrm{2}}}} \right)}^{\mathrm{2}}\Big/\nu.
\end{eqnarray*}

Here, d represents the slit clearance, *u_x_* is the maximum flow velocity within the slit, and *ν* is the kinematic viscosity of the fluid. The calculation is conducted for the slit position between Baffle C and the upper surface of the sample at the cross-section y = 100 mm (Fig. 1e). As shown in Table 2, when d ≥ 10 mm, *L_e_* > 0.5 mm. The boundary layer thickness within the undeveloped region at a distance of 0.5 mm from the slit entrance can be calculated using Equation (2) derived from the Navier–Stokes equations [50,51]:


(2) \begin{eqnarray*}
\frac{{\nu {\mathrm{x}}}}{{{u}_x{{\left( {\frac{{\mathrm{d}}}{{\mathrm{2}}}} \right)}}^{\mathrm{2}}}} = \frac{{\mathrm{1}}}{{{\mathrm{10}}}} \! \left[ {\frac{{{\mathrm{2\delta }}}}{{\mathrm{d}}}{\mathrm{7 + 48ln}}\left( {{\mathrm{1 - }}\frac{{\mathrm{1}}}{{\mathrm{3}}}\frac{{{\mathrm{2\delta }}}}{{\mathrm{d}}}} \right) + \frac{{{\mathrm{27}}\frac{{{\mathrm{2\delta }}}}{{\mathrm{d}}}}}{{{\mathrm{3 - }}\frac{{{\mathrm{2\delta }}}}{{\mathrm{d}}}}}} \! \right],
\end{eqnarray*}

where δ is the boundary layer thickness and *x* represents 0.5 mm.

**Table 2.** The ratio of the boundary layer thickness to the slit clearance.

d (mm)	10	12	14	16
*L_e_* (mm)	0.75	0.86	0.95	1.08
δ (mm)	4.38	5.03	5.66	6.20
δ/d (%)	43.85	41.90	40.47	38.72

The calculation results indicate that as the slit clearance d increases, the boundary layer thickness also increases; however, the proportion of boundary layer thickness to slit clearance gradually decreases (Table 2). Based on the definition of a boundary layer, the fluid velocity within the boundary layer is lower than that outside it, allowing for a longer residence time of the precursor near the sample surface, resulting in a more complete reaction and higher precursor utilization. Results of –OH surface coverage at different slit clearance d after adding Baffles A, B and C indicate that when d = 10 mm, –OH surface coverage is minimized, effectively increasing precursor utilization (Fig. S8).

The ratio of boundary layer thickness to slit clearance is closely related to the extent of the surface reaction. The TMA concentration gradient along the *z*-axis (Fig. 1e) at the sample surface reflects the TMA consumption during the surface reaction. A greater concentration gradient indicates a higher rate of TMA consumption at that location, while a smaller gradient suggests lower consumption. With Baffles A, B and C and a slit of d = 10 mm, three points are selected within the slit at the centerline of Baffle C (Fig. 1e) to calculate the TMA concentration gradient and its temporal variation. Points p, o and q are positioned at distances of ^2^/_3_d, ^1^/_3_d and directly on the surface, respectively. Here, p is located outside the boundary layer, while o and q are within the boundary layer. The temporal changes in concentration gradients at these three points (Fig. 1e) reveal that concentration gradients within the boundary layer are higher than those outside, indicating that the surface reaction primarily consumes precursor within the boundary layer near the surface. TMA concentration at points p, o and q first increases and then decreases over time (Fig. S14). Point p exhibits the highest concentration peak and reaches it in the shortest time, while point q shows the lowest concentration peak and takes the longest time to reach it. This is due to the low carrier gas velocity within the boundary layer, which allows for more extensive interaction with surface –OH vacancy sites, thereby promoting efficient surface reactions.

Within the boundary layer, the primary mechanism of precursor mass transfer is diffusion. The Péclet number (*Pe*) is used to represent the relative contributions of convective and diffusive mass transfer; when *Pe* > 1, convective transfer dominates, while *Pe* < 1 indicates that diffusion is the main mode of mass transfer [52]. The dominant mass transfer mechanism of the precursor within the slit (denoted as Region S, located between Baffle C and the sample upper surface) along the centerline of Baffle C is influenced by the boundary layer thickness (Fig. 1e). At *t* = 2.5 s, the concentration gradients across this region reach their peak values, maximizing the mass transfer rate (Fig. 1e). With Baffles A, B and C and d = 10 mm, the Region S where *Pe* < 1 along the *z*-axis at a purge time of *t* = 2.5 s is dominated by diffusive mass transfer (Fig. 1e; the *Pe* distribution for other d is provided in Fig. S10). Along the *z*-axis (Fig. 1e, Fig. S10c), the thickness of the *Pe* < 1 region also increases with d, and this thickness is comparable to the boundary layer thickness previously calculated. However, its proportion relative to d decreases (the ratio of the thickness of the *Pe* < 1 region to d is smaller with the installation of baffles compared to when no baffles are used, Fig. S11), indicating that diffusion remains the primary mode of mass transfer along the *z*-axis within the boundary layer, facilitating surface reactions.

**Figure 2.**
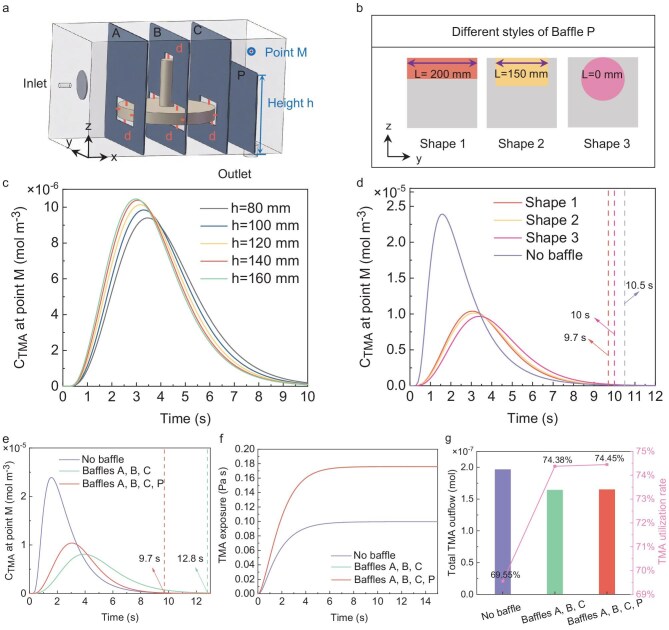
Optimization after the addition of purge Baffle P. (a) Relative position of the Baffle-OptCombo to the showerhead-shaped sample. (b) Different styles of Baffle P. (c) TMA concentrations at point M at different baffle heights. (d) Concentrations of TMA at point M with different baffle shapes for a certain exposure area. (e) TMA concentrations and (f) TMA exposure at point M at different baffle numbers. (g) Outflow at the outlet and utilization of TMA at different baffle numbers.

The addition of baffles within the chamber inevitably increases precursor purge time. To evaluate the purge effectiveness, the purge time at point M, located directly above the chamber outlet, is used as an indicator. The concentration profile at point M (Fig. 2a) shows that adding Baffles A, B and C increases the purge time by 3.1 s compared to no baffle configuration. To reduce the purge time without wasting the carrier gas, and considering the difficulty of pressure control in the experiment, an additional Baffle P is introduced between Baffle C and the chamber outlet to accelerate the purge process (Fig. 2a). The height and shape of Baffle P impact the purge time. The effects of altering the shape of Baffle P (gray section) and the exposed cross-sectional shape (colored section) on purge time are investigated (Fig. 2b). For rectangular baffle Shape 1, changes in TMA concentration over time at point M at different heights indicate that increasing the height gradually reduces purge time. When the height (denoted as h) exceeds 140 mm, however, further height increases do not significantly reduce purge time, so h = 140 mm is selected (Fig. 2c). With a constant exposed area, the shape is modified to Shape 2 and Shape 3 (Fig. 2b). Table 3 presents the top exposed length L and hydraulic diameter for each shape. Results of TMA concentration changes at point M over time for different exposed shapes indicate that the purge time for Shape 1 matches that of the no-baffle configuration at this point, while Shape 2 and Shape 3 require longer purge times (Fig. 2d). The –OH surface coverage of the object after the addition of Baffle P remained unchanged compared to that observed after the installation of Baffles A, B and C (Fig. S9). In summary, a larger top exposed length L and smaller hydraulic diameter of the exposed shape in Baffle P are advantageous for efficient purging. The optimal baffle combination (denoted as Baffle-OptCombo) is the addition of Baffles A, B, C and P, with d = 10 mm, Baffle P in Shape 1, and h = 140 mm.

**Table 3.** Comparison of different baffle shapes.

Style of Baffle P	Shape 1	Shape 2	Shape 3
Length (mm)	200	150	0
Hydraulic diameter (mm)	52	63	87

The simulation results further demonstrate that the addition of Baffle P not only improves purge efficiency but, more importantly, ensures that the surface reaction is not affected. The TMA concentration at point M indicates that adding Baffles A, B and C extends the purge time, while the inclusion of Baffle P reduces the purge time to 9.7 s, making it identical to the no-baffle condition (Fig. 2e). When the pulse frequency exceeds this threshold—meaning the purge time falls below 9.7 s—non-ideal ALD deposition occurs within the chamber, and achieving the ideal deposition quality through baffle design alone becomes unattainable. Conversely, if the pulse frequency remains below this critical value, sufficient purge time is maintained, enabling optimal baffle design without compromising the uniformity and quality of the deposition. Exposure, defined as the integral of partial pressure over time, is an indicator of whether the precursor amount during the ALD process is sufficient for surface saturation adsorption. Expressed in Langmuir units (1 L = 10^6^ Torr s = 7500 Pa s) [53], it effectively represents changes in precursor concentration within the boundary layer. The addition of Baffles A, B and C increases precursor exposure on the sample surface (Fig. 2f), while the maximum –OH surface coverage of the object decreases (Fig. S13). Meanwhile, the inclusion of Baffle P does not affect surface exposure and the maximum –OH surface coverage, further confirming that Baffle P has no impact on surface reaction of the sample (Fig. 2f, Fig. S13). With the addition of Baffle-OptCombo, the TMA outflow at the chamber outlet decreased, leading to an increase in precursor utilization from 69.55% to 74.45% (Fig. 2g). This enhancement can be attributed to the carefully designed positioning of the baffles, which are placed in regions that exhibit high –OH surface coverage in the absence of the baffles. Thus, identifying regions with high –OH surface coverage is essential for determining the optimal placement of the baffles. The Baffle-OptCombo decreases precursor waste on the complex surfaces of the sample, while ensuring that the purge time remains unchanged.

While the baffle design principle proposed by this study may not guarantee the microscopic film step coverage into high-aspect-ratio trenches or multi-scale porous structures, the slitted baffles will provide an effective method to control the precursor distribution in the boundary layer around other macroscopic 3D complex objects. When the shape of the deposited 3D object changes (from showerhead configuration to other large-scale complex geometries), simulations could be conducted to identify regions with high –OH surface coverage, where slitted baffles could be added. The slit clearance between the baffles and the object surface should be carefully designed to regulate the ratio of boundary layer thickness to slit clearance, which ensures maximum precursor concentration within the boundary layer, enhancing mass transfer to the surface while minimizing surface –OH surface coverage. This approach may enhance deposition uniformity, precursor utilization, with accelerated purge duration.

**Figure 3.**
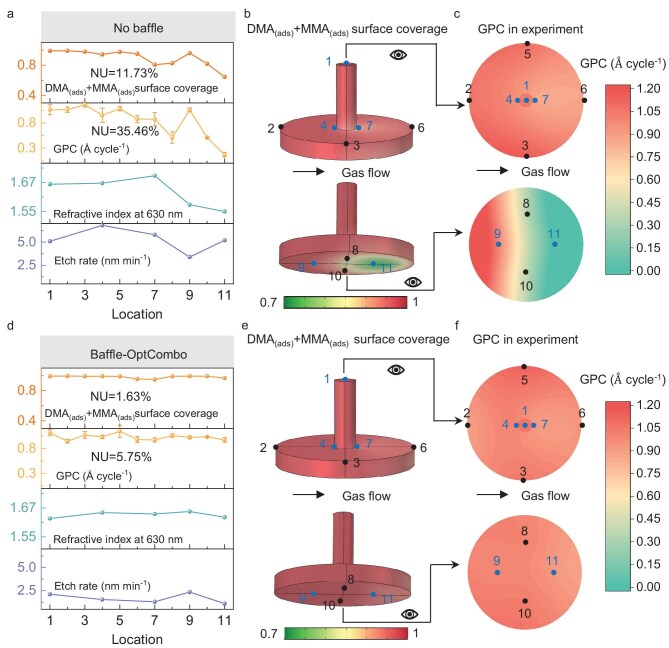
Effect of addition of baffles on deposition uniformity: DMA_(ads)_ + MMA_(ads)_ surface coverage, GPC in experiment, refractive index of films and etch rate in plasma at different positions of the complex sample: (a) without baffle and (d) with Baffle-OptCombo; (e) DMA_(ads)_ + MMA_(ads)_ surface coverage map in simulation and positions of the eleven points on the sample surface: (b) without baffle and (e) with Baffle-OptCombo. The GPC distribution across 11 experimental points: (c) without baffle and (f) with Baffle-OptCombo. Five blue points (Points 1, 4, 7, 9 and 11) are representative positions with a significant impact on deposition uniformity, while the remaining six black points serve as reference points for spatial distribution analysis.

**Figure 4.**
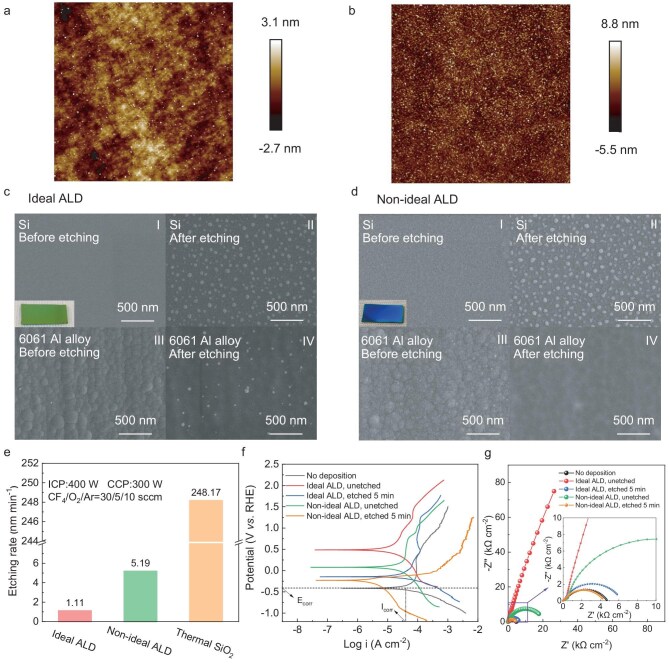
Electrochemical and surface analysis of Al_2_O_3_ films on 6061 aluminum alloy and silicon substrate: AFM of (a) ideal ALD and (b) non-ideal ALD deposited film at Position 11 of the complex 3D sample; SEM of (c) ideal ALD and (d) non-ideal ALD deposited film at Position 11 of the complex 3D sample: (I) 20 nm Al_2_O_3_ films on silicon substrate, (II) after 5 min fluorinated plasma etching, (III) 20 nm Al_2_O_3_ films on 6061 aluminum alloy substrate and (IV) after 5 min fluorinated plasma etching. (e) Etching rate of ideal ALD, non-ideal ALD deposited film and thermal SiO_2_ film. (f) Polarization curves and (g) Nyquist plots of 6061 aluminum alloy samples with different Al_2_O_3_ coatings.

To verify the effectiveness of the Baffle-OptCombo in improving the deposition uniformity of the sample, experiments were conducted with and without the baffles, using the growth per cycle (GPC) at 11 positions as the performance indicator (detailed position in Fig. 3b and e; Fig. S19), which is compared to the –OH surface coverage obtained from the simulation. Both the sample and baffles were made of 6061 aluminum alloy, with the baffles covered by PTFE tapes. XPS signal confirmed that the adsorption of TMA on the PTFE tape surface is substantially lower than on aluminum alloy (Table 1; Fig. S18), indicating that the consumption of the precursor by the baffles is negligible. Al_2_O_3_ deposition was conducted under the same pulse and purge time conditions used in the simulation with and without baffles: TMA and H_2_O dose times were 0.02 and 0.01 s, respectively, with corresponding purge times of 10 and 15 s, which was denoted as the best-known method (BKM) recipe of this study. With the addition of the Baffle-OptCombo, the GPC of every position increased to approximately 1 Å cycle^−1^, reflecting an ideal ALD growth process, while thickness NU decreased from 35.46% to 5.75% (Fig. 3a and d). This improvement corresponds to a reduction in the NU of surface coverage for the two by-products—dimethylaluminum [DMA_(ads__)_] and monomethylaluminum [MMA_(ads__)_] (Section S1.2)—from 11.73% to 1.63% in the simulation (Fig. 3b and e). The discrepancies observed between the simulation and experiments may be attributed to fluctuations in factors such as obtained from the simulation, chamber pressure and the slight adsorption of precursors on the baffles. The GPC contour maps for both the top and bottom surface visually highlight a more uniform deposition following the baffle installation (Fig. 3c and f; the five Positions 1, 4, 7, 9 and 11 in blue represent the key points that affect surface uniformity). Moreover, the consistent refractive index (measured using an ellipsometer) across the surface after adding baffles indicates a uniform film density distribution (Fig. 3a and d), which also serves as an indirect measure of deposition uniformity [54,55]. The AFM results of the films deposited on the silicon substrate at Position 11 show that the average surface roughness (R_a_) decreased from 1.4 to 0.628 nm after the addition of the Baffle-OptCombo, indicating a smoother surface (Fig. 4a and b). This, combined with the increase in refractive index and GPC approaching 1 Å cycle^−1^ at Position 11, demonstrates that the films deposited after the baffle addition are denser, reflecting an ideal deposition. In summary, adding baffles improves both precursor utilization and surface deposition uniformity.

To directly evaluate the plasma etching resistance of different quality films, silicon coupon samples (7 × 15 mm^2^) were attached at five key positions on the sample surface (blue points: Positions 1, 4, 7, 9 and 11 in Fig. 3b and e, where Positions 9 and 11 are located at the quarter points along the diameter of the bottom surface of the object (details of the other positions can be found in Fig. S19), for which the film thickness was measured using spectroscopic ellipsometry as an indicator for the thickness on aluminum alloy. Using the aforementioned BKM recipe, Al_2_O_3_ was deposited for 200 cycles, both with and without the baffles. The deposited Al_2_O_3_ films were then subjected to fluorine plasma etching in an inductively coupled plasma-capacitively coupled plasma (ICP-CCP) system. The gas flow rates were CF_4_/O_2_/Ar = 30/5/10 sccm, with an ICP power of 400 W, a CCP power of 300 W, and a chamber pressure of 38 Pa. Scanning electron microscopy (SEM) images reveal different surface morphologies on each substrate deposited at Position 11 of the 3D sample following 200 cycles of Al_2_O_3_ deposition. The surface of the silicon coupon sample under ideal ALD conditions was smooth, while the aluminum alloy substrate showed visible grain formations (Fig. 4c). For non-ideal ALD conditions, the silicon surface remained smooth, but larger grains were observed on the aluminum alloy surface compared to the ideal ALD sample (Fig. 4d). After 5 minutes of plasma etching, small particles appeared on the silicon surface, and the grain structure on the aluminum alloy surface disappeared. Cross-sectional SEM images (Fig. S16) confirm that while the grain structures disappeared, the Al_2_O_3_ film was not fully etched away. The etching rate results of films at different positions on the surface of 3D sample, with and without the baffles, show that the etching rate of films deposited without baffles is higher than that with Baffle-OptCombo (Fig. 3a and d). Position 11 (where the –OH coverage is highest in the simulation and the growth rate is slowest in the experiment) exhibits the poorest film quality and the highest etching rate. Specifically, the etching rate of thermally grown SiO_2_ (248.17 nm min^−1^) is significantly higher than that of ALD Al_2_O_3_, while the non-ideal Al_2_O_3_ film at Position 11 (5.19 nm min^−1^) exhibits an etch rate nearly five times faster than the ideal sample at the same position (1.11 nm min^−1^) (Fig. 4e). This confirms that the introduction of carefully designed baffles indeed enables the growth of an ideal ALD film with excellent plasma etching resistance.

To compare the protective performance of ALD films with different levels of quality, a 2 × 2 cm^2^ aluminum alloy sample was placed at Position 11 and deposited under different conditions (Fig. 3b and e). Using our BKM recipe with the Baffle-OptCombo, an ideal Al_2_O_3_ film was deposited, achieving a GPC of 1.13 Å cycle^−1^, close to the ideal GPC of 1 Å cycle^−1^, which results in a final thickness of 340 nm after 3000 cycles, followed by 5 min of plasma etching. To deposit the low-quality Al_2_O_3_ film with a comparable 340 nm thickness, with a low GPC at Position 11 without baffles (0.086 Å cycle^−1^ with our BKM recipe, corresponding to high –OH coverage in the simulation) within an acceptable total deposition time, a modified deposition recipe was developed by increasing both TMA and H₂O dose durations to 0.1 s and reducing the purge time to 8 s. Electrochemical tests, including polarization curves and electrochemical impedance spectroscopy (EIS), were conducted to assess the corrosion performance of the films. In the polarization curve test, higher corrosion potential (E_corr_) and lower corrosion current density (I_corr_) indicate better corrosion resistance [13,56–58]. The ideal ALD deposited sample exhibits superior corrosion resistance, with higher E_corr_ and lower I_corr_ compared to the non-ideal ALD deposited sample (Fig. 4f, Table S5). The corrosion protections of ideal ALD-Al_2_O_3_ could be further confirmed by EIS, where the coating significantly increases the charge transfer resistance compared to non-ideal ALD sample, as indicated by the enlarged semicircle diameter in the Nyquist plots (Fig. 4g, Table S6) [59–61]. Furthermore, the plasma-etched ideal ALD-deposited sample exhibits superior corrosion resistance, with higher E_corr_, lower I_corr_ and large capacitive arc radius compared to the undeposited one (Fig. 4f and g; Table S6, Fig. S21), demonstrating that the films retained some corrosion resistance even after etching. The electrochemical results further validate the simulation, showing that the reduced surface –OH coverage, or increased DMA_(ads)_+MMA_(ads)_ surface coverage, in simulation at Position 11 indeed leads to more ideal deposition upon the introduction of the slitted baffles, thereby experimentally demonstrating enhanced etch resistance. In summary, films deposited with the baffles demonstrated excellent quality and superior resistance to plasma etching.

## CONCLUSION

This paper describes the design and implementation of a feasible approach to increase the precursor concentration and exposure within the boundary layer in ALD for coating complex 3D objects, which is enabled by introduction of slitted baffles within the chamber. Under conditions of inadequate precursor, baffles are introduced as internal components to enhance mass transfer between the precursor and the object surface. –OH surface coverage is used as an indicator of the extent of surface adsorption and NU of films. Simulation results determined that increasing the ratio of boundary layer thickness to slit clearance significantly enhances precursor adsorption on the surface, leading to improved film uniformity and quality, accompanied by an increase in precursor utilization from 69.55% to 74.45%. Under the optimal baffle configuration, the simulation showed a decrease in NU of the DMA_(ads)_ and MMA_(ads)_ surface coverage from 11.73% to 1.63%, while experimental results demonstrated a reduction in film thickness NU from 35.46% to 5.75% across eleven representative points over a showerhead-like sample, highlighting a significant improvement in film uniformity on 3D objects. For the purge baffle, a smaller hydraulic diameter of the exposed shape between the baffle and chamber facilitates more effective purging, reducing the purge time from 12.8 to 9.7 s. Moreover, the Al_2_O_3_ film deposited by ideal ALD exhibits a low etch rate of 1.11 nm min^−1^, five times more resistant than that of films deposited under non-ideal ALD conditions (5.19 nm min^−1^), demonstrating the critical importance of ensuring ideal ALD deposition across all surfaces of a 3D object surface for enhancing its resistance to plasma etching.

## METHODS

### ALD of Al_2_O_3_ thin film

The ALD experiments were conducted in a custom-built facility (dimensions: 350 mm × 200 mm × 200 mm), where a complex 3D object (showerhead-like sample made of Φ165/20 mm disk with a Φ25/80 mm rod) and baffles, made from 6061 aluminum alloy and wrapped in PTFE tape, were assembled in the ALD chamber (details in Fig. S17). Argon was used as the purge gas, with a flow rate set to 400 sccm by a mass flow controller (Sevenstar CS200A), and an equilibrium pressure of 170 Pa was maintained in the chamber. Deposition was performed at 150°C, while both TMA and water were held at room temperature (25°C). Based on the Antoine equation, the vapor pressures of TMA and water at 25°C are 1294 and 3149 Pa, respectively, both of which exceed the baseline pressure in the chamber of 170 Pa. When the precursor valve opens, this pressure difference drives the precursor into the chamber. The dose time is carefully controlled to regulate the precursor volume entering the chamber. The thickness and the refractive index of the thin films deposited on silicon wafers, positioned on the surface of the 3D sample, were measured using an M-2000DI ellipsometer (J. A. Woollam), with the angle of incidence set between 60° and 70°. As Al_2_O_3_ is a transparent thin film, the Cauchy model was employed to fit both the film thickness and the wavelength-dependent refractive index through the optical parameter A_n_ in the transparent film equation.

### Modeling

To determine the optimal configuration of internal components, finite element analysis (FEA) was used to simulate fluid dynamics within the system. The simulation examined four key sections: fluid transfer, heat transfer, mass transfer and surface reaction. Both steady-state and transient-state conditions were modeled to simulate laminar flow within the chamber, as well as mass transfer dynamics when the valve opened and closed. The chemical properties of different substances are listed in a supplementary table (Table S2). Optimization was performed for varying numbers of baffles and slit clearance d to identify the most effective baffle configuration. Additionally, grid independence verification was performed to ensure the accuracy of the simulation results (Fig. S15). Further details are provided in the supplementary information (Section S1).

### Electrochemical testing and characterization

Deposition samples were placed at five strategic points on the surface of the 3D object (Fig. 3e). Plasma etching experiments were then conducted using a custom-built ICP-CCP system. Al_2_O_3_ thin films were deposited onto both polished silicon (100) and 6061 aluminum alloy substrates, followed by thickness and electrochemical measurements. Polarization curves and electrochemical impedance tests were conducted in a 1 M Na_2_SO_4_ solution (Fig. S20). Surface morphology before and after plasma etching was examined using field emission SEM. SEM imaging was performed with a Regulus 8100, operating at an accelerated voltage range of 0.1–30 kV, with a secondary electron resolution of 0.8 nm at 15 kV and 1.1 nm at 1 kV. Further details are provided in the supplementary information (Section S2).

## Supplementary Material

nwaf247_Supplemental_File
