# Supplementary material for: Deposition of uniform films on complex 3D objects by atomic layer deposition for plasma etch-resistant coatings
Source: Natl Sci Rev. 2025 Jun 17;12(8):nwaf247. doi: 10.1093/nsr/nwaf247 (PMC12281629; doi:10.1093/nsr/nwaf247)
Supplement: nwaf247_Supplemental_File [file nwaf247_supplemental_file.pdf]

# Supporting Information

# Deposition of Uniform Films on Complex 3D Objects by Atomic Layer Deposition for Plasma Etch Resistant Coatings

Xin Han[1,2], Yixian Wang[1,2], Yumo Tian[1,2], Yafeng Wang[1,9], Lipei Peng[9], Chunlei Pei[1,2,3,4,5,6,7], Tuo Wang[1,2,3,4,5,6,7,*] and Jinlong Gong[1,2,7,8,*]

[1]School of Chemical Engineering & Technology, Key Laboratory for Green Chemical Technology of Ministry of Education, Tianjin University; Collaborative Innovation Center for Chemical Science & Engineering; Tianjin 300072, China;

[2]International Joint Laboratory of Low-carbon Chemical Engineering of Ministry of Education, Tianjin 300350, China;

[3]National Industry-Education Platform of Energy Storage, Tianjin University, Tianjin 300350, China;

[4]Haihe Laboratory of Sustainable Chemical Transformations, Tianjin 300192, China;

[5]Joint School of National University of Singapore and Tianjin University, International Campus of Tianjin University, Fuzhou 350207, China;

[6]Zhejiang Institute of Tianjin University Ningbo, Zhejiang 315201, China;

[7]State Key Laboratory of Synthetic Biology, Tianjin University, Tianjin 300072, China;

[8]Tianjin Normal University, Tianjin 300387, China;

[9]Peric Special Gases Co., Ltd, Handan 056002, China

***Corresponding authors**. E-mails: jlgong@tju.edu.cn; wangtuo@tju.edu.cn

Keywords: atomic layer deposition, thin film, film uniformity, 3D object deposition, plasma etching, etch resistant coating

## Nomenclature

$k_B$: Boltzmann constant $1.38\times10^{-23}$, [J $K^{-1}$]

$T$: Temperature, [K] or [°C]

$K_n$: Knudsen number, [-]

$P$: Static pressure, [Pa]

$L$: Characteristic length of the reactor, [m]

$u$: Local flow velocity [m $s^{-1}$]

$Re$: Reynolds number, [-]

$Sc$: Schmidt number, [-]

$\vec{u}$: Velocity vector, [m $s^{-1}$]

$\bar{\bar{I}}$: Unit tensor, [-]

$\vec{g}$: Gravitational acceleration vector, [m $s^{-2}$]

$c_p$: Specific heat of fluid, [J $mol^{-1}$ $K^{-1}$]

$\vec{J_i}$: Diffusion flux vector, [kg $m^{-2}$ $s^{-1}$]

$j_i$: Diffusion flux, [kg $m^{-2}$ $s^{-1}$]

$k$: Mixture thermal conductivity, [W $m^{-1}$ K]

$M$: Molecule weight, [kg $mol^{-1}$]

$\omega_i$: Mass fraction of species i, [-]

$P_r$: Prandtl number

$\vec{q}$: Heat flux

$h$: Planck constant

$\Delta G_i$: Gibbs activation free energy for the reaction pathway i

$Pe$: Péclet number

***D:*** Mass diffusion coefficient

***Greek letters***

***ε*: Lennard-Jones energy parameter, [J per molecule]

***λ*: Mean free path, [m]

***μ*: Dynamic viscosity, [Pa s]

***ρ*: Density, [kg $m^{-3}$]

***σ*: Lennard-Jones collision diameter, [m]

# Contents

1. Supplementary Texts of Simulation

2. Supplementary Texts of experiment

3. Supplementary Tables

4. Supplementary Figures

5. References

# S1 Supplementary Texts of Simulation

## S1.1 Governing equations and model assumptions

The fluid dynamics within the ALD chamber are influenced by its distinct dimensions, along with the temperature and pressure of the gas, thereby requiring the application of relevant governing equations.

In the kinetic theory of gases, the mean free path of a particle($\lambda$), such as a molecule, is defined as the average distance the particle travels before colliding with other moving particles [1].

$$\lambda=\frac{k_B T}{\sqrt{2}\pi\sigma^2 P} \quad \text{S1}$$

The Knudsen number can be defined by comparing the mean free path to the characteristic length of the system.[1]

$$Kn=\frac{\lambda}{L} \quad \text{S2}$$

The Boltzmann constant, $k_B$= 1.38×10$^{-23}$ J K$^{-1}$, and the maximum deposition temperature $T$ = 423 K are used in the calculations. λ represents the mean free path of the gas mixture, and L denotes the characteristic length of the ALD equipment. The molecular diameters of argon (Ar) and trimethylaluminum (TMA) are $\sigma_{Ar}$ =340 pm and $\sigma_{TMA}$ =530 pm, respectively. The minimum chamber pressure is $P$ = 170 Pa, and the inlet characteristic length of the chamber is d = 0.0075 m. Based on the equation of S2, the maximum Knudsen number is calculated to be 0.00384 < 0.01, where the assumptions of a continuous medium can be employed to simulate the flow using the Navier-Stokes equations with a no-slip boundary condition.

In fluid mechanics, the Reynolds number (*Re*) is a dimensionless quantity used to predict the flow regime of a fluid, defined as the ratio of inertial forces to viscous forces [2].

$$Re=\frac{\rho uL}{\mu} \tag{S3}$$

At the inlet, the fluid velocity is at its maximum; therefore, the inlet diameter is chosen as the characteristic length. When $T$ = 383 K, the Reynolds number *Re* is calculated to be 31.57. This result indicates that the flow is in the laminar regime during the ALD deposition process.

In the computational fluid dynamics (CFD) model, the heat transfer process of the fluid is also considered, where heat is transferred between the fluid and the chamber wall, as well as between the fluid and the surfaces of irregular objects, through convective heat transfer. The fluid in this model is assumed to be compressible, continuous, and ideal.

When considering mass transfer, since the concentration of the precursor entering the chamber during the pulse is much lower than that of the carrier gas, the mass transfer of the precursor is simplified as a trace species transfer. The model can be divided into two stages: during the purge of the precursor, the system is considered to be in a steady-state process; whereas, at the moment the precursor pulse enters the chamber, the valve opens, causing the chamber pressure to rise, and this process is treated as a transient process.

In continuum mechanics, the Péclet number (*Pe*) is a dimensionless number that plays a significant role in analyzing transport phenomena within a continuum [2]. It is defined as the ratio between the advection rate of a physical quantity by the flow and the diffusion rate of the same quantity due to an associated gradient. For mass or species transfer, the Péclet number is given by the product of the Reynolds number and the Schmidt number ($Re \times Sc$).

$$Pe=\frac{\text{advective transport rate}}{\text{diffusive transport rate}}=\frac{\mathrm{Lu}}{\mathrm{D}}=\mathrm{ReSc} \qquad \text{S4}$$

where $D$ is the mass diffusion coefficient, $u$ is the local flow velocity and $L$ is characteristic dimension. If $Pe > 1$, convective mass transfer dominates the mass transfer process, and the influence of diffusion is minimal. Conversely, if $Pe < 1$, diffusion becomes the primary mode of mass transfer.

The transport phenomena within the ALD reactor are governed by equations for mass, momentum, and energy conservation, coupled with chemical species conservation, as outlined below:

**Continuity equation** [2]**:**

$$\frac{\partial \rho}{\partial t}+\nabla\cdot(\rho\vec{u})=0 \qquad \text{S5}$$

**Navier-Stokes equation** [2]**:**

$$\frac{\partial(\rho\vec{u})}{\partial t}+\nabla\cdot(\rho\vec{u}\vec{u})=-\nabla P+\nabla\cdot\left[\mu\left(\nabla\vec{u}+\nabla\vec{u}^{T}\right)-\mu\frac{2}{3}(\nabla\cdot\vec{u})\bar{\bar{I}}\right]+\rho\vec{g} \qquad \text{S6}$$

**Energy equation** [2]**:**

$$C_p\frac{\partial(\rho T)}{\partial t}+C_p\nabla\cdot(\rho\vec{u}T)=\nabla\cdot(k\nabla T) \qquad \text{S7}$$

**Species transport equation** [2]**:**

$$\frac{\partial(\rho y_i)}{\partial t}+\nabla\cdot\left(\rho\vec{V}y_i\right)=-\nabla\cdot\vec{j}_i \qquad \text{S8}$$

The diffusion flux is calculated [2]:

$$j_i = -\rho\omega_i \sum_{k=1}^{n-1} D_{ik} \left[ \nabla x_k + (x_k - \omega_k) \frac{\nabla p}{P} \right] - D_{T,i} \frac{\nabla T}{T} \quad \text{S9}$$

Here, $D_{ik}$ denotes the Maxwell-Stefan diffusion coefficient, and $x_k$ represents the mole fraction. Equation (S9) illustrates Fick's law, accounting for the Soret effect under constant mixture composition. The thermal diffusion coefficients are calculated as follows [2]:

$$D_{T,i} = -2.95\times10^{-7} T^{0.659} \left[ \frac{M_i^{0.511} x_i}{\sum_{i=1}^{n} M_i^{0.511} x_i} - \omega_i \right] \cdot \left[ \frac{\sum_{i=1}^{n} M_i^{0.511} x_i}{\sum_{i=1}^{n} M_i^{0.489} x_i} \right] \quad \text{S10}$$

**Transport properties**

The Chapman-Enskog theory gives expressions for the transport properties in terms of the intermolecular potential energy φ(r):[2]

$$\varphi(\mathrm{r}) = 4\varepsilon \left[ \left(\frac{\sigma}{r}\right)^{12} - \left(\frac{\sigma}{r}\right)^{6} \right] \quad \text{S11}$$

where $r$ denotes the collision distance, $\sigma$ is the characteristic molecular diameter, often referred to as the collision diameter, and $\varepsilon$ is the characteristic energy, which is the maximum attractive energy between a pair of molecules.

When $\sigma$ and $\varepsilon$ are unknown, they can be estimated based on the properties of the fluid at the critical point (c) [2].

$$\frac{\varepsilon}{k} = 0.77 T_c \quad \text{S12}$$

$$\sigma = 2.44 \left(\frac{T_c}{P_c}\right)^{\frac{1}{3}} \quad \text{S13}$$

Here ε/k and $T$ are in K, $\sigma$ is in Angstrom units (1 Å = $10^{-10}$ m), $P_c$ is in atmospheres.

The viscosity of a pure monatomic gas with molecular weight M can be expressed in terms of the Lennard-Jones parameters as follows [2]:

$$\mu=2.6693\times10^{-5}\frac{\sqrt{MT}}{\sigma^2\Omega_\mu} \tag{S14}$$

$$\Omega_\mu=\frac{1.16145}{T^{*0.14874}}+\frac{0.52487}{exp(0.77320T^*)}+\frac{2.16178}{exp(2.43787T^*)} \tag{S15}$$

$$T^*=kT/\varepsilon \tag{S16}$$

Here, *M* is its molecular mass, *T* is temperature and the dimensionless quantity $\Omega_\mu$ is a slowly varying function of the dimensionless temperature *Tk/ε*, with an order of magnitude of unity.

### S1.2 Surface chemistry

This paper discusses the process of depositing $Al_2O_3$ thin films using TMA and water as precursors. The ALD deposition of aluminum oxide thin films consists of two stages: the TMA pulse and the water pulse. This study focuses on improving the utilization efficiency of TMA, and therefore, only the surface reactions during the TMA pulse stage are considered.

**TMA pulse surface reaction equation** [3]**:**

TMA adsorbs reversibly onto the surface OH group:

$$Al(CH_3)_{3(g)}+OH_{(s)}\leftrightarrow H\text{-}O\text{-}Al(CH_3)_{3(ads)} \tag{R1}$$

where the species $H\text{-}O\text{-}Al(CH_3)_{3(ads)}$ is referred to as $TMA_{(ads)}$. Upon adsorption onto the -OH site, the TMA molecule may either desorb or participate in a reaction. In this reaction, a $CH_3$ group

from TMA reacts with the hydrogen atom of the hydroxyl group, leading to the formation of $CH_4$, which is released as a gaseous byproduct:

$$TMA_{(ads)} \rightarrow O\text{-}Al(CH_3)_{2(s)} + CH_{4(g)} \qquad R2$$

where the $O\text{-}Al(CH_3)_{2(s)}$ surface species will subsequently be designated as dimethyl aluminum, or $DMA_{(ads)}$. Density functional theory calculations have shown that a DMA molecule can undergo a secondary reaction with a neighboring -OH site on the surface, resulting in the release of $CH_4$ as a gaseous byproduct:

$$DMA_{(ads)} \rightarrow\rightarrow O_2\text{-}Al(CH_3)_{(s)} + CH_{4(g)} \qquad R3$$

where the $O_2\text{-}Al(CH_3)_{2(s)}$ surface species will be referred to as monomethyl aluminum, or $MMA_{(ads)}$, from this point onward.

R3 and R4 can be combined as follows:

$$2TMA_{(ads)} \rightarrow DMA_{(ads)} + MMA_{(ads)} + 3CH_{4(g)} \qquad R4$$

Based on the reaction process outlined above, two TMA molecules react with three hydroxyl groups. In our computational model, each TMA molecule adsorbs onto an average of 1.5 -OH groups, resulting in a site occupancy number of 1.5 for the $TMA_{(ads)}$ species. When each adsorbed TMA molecule reacts, it yields equal amounts of $DMA_{(ads)}$ and $MMA_{(ads)}$ species, where $DMA_{(ads)}$ has a site occupancy number of 1 and $MMA_{(ads)}$ has a site occupancy number of 2.

**Implementation of the surface chemistry**[3]

This section outlines the specific mechanisms incorporated into the surface chemistry model.

For the TMA adsorption step (R1), the adsorption rate is denoted by $R_{ads}$, where $i$ represents the adsorbed species:

$$R_{ads,i}=s_i \cdot Flux_i \qquad \text{S17}$$

where $s_i$ is the sticking coefficient of TMA and $Flux_i$ is the molar flux of the gaseous species TMA.

$$s_i=s_{0,i} \cdot \theta_a \cdot e^{-\frac{\Delta G_{ads,i}}{k_B T}} \qquad \text{S18}$$

The adhesion coefficient ($s_i$) is related to the steric hindrance effect. The initial adhesion coefficient is defined as $s_{0,i}$, and the initial available adsorption sites ($\theta$) correspond to the initial coverage of surface OH groups, $C_{OH,0}$, which is temperature-dependent. The OH surface concentration and the initial adhesion coefficient at a deposition temperature of 150°C are listed in Table S2. The instantaneous number of surface vacancies is represented by $\theta_a$, and $\Delta G_{ads,i}$ refers to the adsorption activation energy.

The molar flux is calculated using the Hertz-Knudsen equation:

$$Flux_i=\frac{P_i}{\sqrt{2\pi M_i R T}} \qquad \text{S19}$$

where $P_i$ is the partial pressure of species $i$, $M_i$ is its molecular mass, $R$ is the ideal gas constant, and $T$ is the temperature. Here, $i$ refers to TMA.

For the $TMA_{(ads)}$ desorption step:

$$R_{des,i}=\frac{k_B T}{h} e^{-\frac{\Delta G_{des,i}}{k_B T}} \cdot c_i \qquad \text{S20}$$

where $R_{des,i}$ indicates the desorption rate of species $i$, $\Delta G_{des,i}$ represents the activation energy for desorption, $k_B$ is the Boltzmann constant, $T$ is the temperature, and $h$ is the Planck constant

For R4, when $TMA_{(ads)}$ undergoes a surface reaction:

$$R_{r,i}=\frac{k_BT}{h}e^{-\frac{\Delta G_{r,i}}{k_BT}}\cdot c_i \qquad S21$$

where $R_{r,i}$ indicates the reaction rate of species $i$, $\Delta G_{r,i}$ represents the activation energy for reaction of species $i$.

**S1.3 Grid independence test**

A commonly used method for solving the partial differential equations in the model construction is the finite element analysis (FEA) method. The main principle of this method is to divide the solution domain into smaller discrete elements, solve the equations approximately within each element, and then combine the solutions of these elements to obtain an approximate solution for the entire domain. This approach simplifies complex problems as much as possible. The discretization of the region is achieved by constructing a mesh, where theoretically, the smaller and denser the mesh, the closer the computed result will be to the true solution. However, in practice, as the mesh size decreases, the computational load increases exponentially. Therefore, while ensuring the accuracy of the results, the computational cost must also be considered. An appropriate meshing strategy is crucial.

In this study, three different mesh configurations were constructed by varying the size and density of the mesh before and after the addition of the baffles to perform a mesh independence verification. The degree of surface reaction in the model is critical for the design of the baffles, with the maximum surface OH coverage serving as the indicator for determining the

appropriateness of the mesh resolution. As the mesh density increases, when the maximum OH coverage on the surface of the three-dimensional object no longer changes, the corresponding mesh configuration is considered optimal, and further mesh refinement becomes unnecessary. When no baffles are added, the optimal mesh size is 306,178 elements, while the optimal mesh size with baffles A, B, C, and P added is 759,755 elements (Fig. S14).

### S1.4 Boundary conditions

The correct boundary conditions are crucial for accurately predicting experimental results using the model. In this model, the inlet mass flow rate is set to 400 sccm, the outlet pressure is 170 Pa, and the fluid at the wall is assumed to be in a no-slip condition. The wall temperature is maintained at 150°C, controlled experimentally using a PID system. The concentration of the precursor entering the chamber at the moment the valve is opened is represented as a time-dependent step function. Immediately upon valve opening, the precursor concentration is set to 0.0075 mol/$m^3$ (estimated based on the pressure increase observed experimentally at the moment the valve opens, using the ideal gas law). After the valve is closed, the precursor flow at the inlet is assumed to be zero.

# S2 Supplementary Texts of Experiment

## S2.1 Films deposition and electrodes preparation

The Baffles A, B, C, P, and complex objects resembling showerhead are all made of 6061 aluminum alloy, with the surfaces of four baffles covered with PTFE tape (Fig. S17). XPS results show that the deposition rate of alumina on the PTFE substrate is significantly lower than 1 Å $cycle^{-1}$ (Table S7). The assembly of baffle plates and complex objects was placed in the center of a batch ALD chamber (Fig. S17), and silicon wafers and aluminum alloy sheets were positioned at eleven key points on their surfaces for deposition (positions shown in Fig. S19). The aluminum alloy sheets were cut to 0.2 mm × 20 mm × 20 mm, sonicated in ethanol for 20 minutes, and then rinsed with deionized water.

The ideal ALD deposition parameters were as follows: TMA dose (0.02 s), Ar purge (10 s), $H_2O$ dose (0.01 s), Ar purge (15 s), with 3000 cycles of deposition. For the non-ideal ALD deposition, the parameters were: TMA dose (0.1 s), Ar purge (8 s), $H_2O$ dose (0.1 s), Ar purge (8 s), with 1500 cycles of deposition. The aluminum alloy sheets deposited using the ideal ALD parameters were then placed in a self-built inductively coupled plasma-capacitively coupled plasma (ICP-CCP) system, where etching was performed for five minutes under mixed gas conditions of $CF_4/O_2/Ar$ at a flow rate of 30/5/10 sccm. The ICP power was 400 W, and the CCP power was 300 W. The etching pressure during the experiment was measured at 38 Pa. Insulating tape was wrapped around four types of aluminum alloy sheets—un-deposited, ideal ALD-deposited, plasma-etched, and non-ideal ALD-deposited—leaving only 1 $cm^2$ uncovered by tape. Different electrodes were then prepared accordingly.

## S2.2 Polarization and impedance curve analysis

The current-voltage polarization curves and electrochemical impedance spectra (EIS) were measured using an electrochemical workstation. The testing system consisted of a three-electrode setup: the reference electrode was a saturated calomel electrode (Ag/AgCl electrode, SCE), the counter electrode was a platinum electrode, and the working electrode was the prepared aluminum alloy sheet. The corrosive solution was a 1 M $Na_2SO_4$ solution, with a pH of 5.52. To ensure the open-circuit potential (OCP) stabilized, the test sample was immersed in the electrolyte for 30 minutes before testing.

For the electrochemical impedance spectroscopy (EIS) measurement, the stable OCP value previously determined was set as the initial voltage. The AC excitation signal had an amplitude of 10 mV, and the scanning frequency range was from 0.01 Hz to 100 kHz.

For the polarization curve measurement, the scan started at a potential of -0.5 mV (relative to the open-circuit potential) and ended at 1 mV (relative to the open-circuit potential), with a scan rate of 0.1 mV $s^{-1}$.

### S2.3 Characterization

The morphology of the samples was characterized by field emission scanning electron microscopy (FE-SEM, HITACHI Regulus 8100, 0.1-30 kV). XPS analysis of the electrodes was performed using a K-Alpha+ system (ThermoFisher Scientific, UK) with an Al K-alpha+ X-ray source. The binding energy was calibrated using the contaminated carbon C 1s peak at 284.80 eV as the reference。

### S2.4 Uniformity measurement

The thickness of $Al_2O_3$ thin film was measured by the M-2000DI ellipsometer (J. A. Woollam). The thickness of the deposited film on the 6061 aluminum alloy surface is considered equivalent to the thickness of the thin film on the silicon wafer surface. We placed samples at eleven key points on the surface (Fig. 3e) of a complex object and calculated the average thickness (mean) and standard deviation (σ). The nonuniformity was calculated as follows:

$$\text{Nonuniformity}=\frac{1\sigma}{\text{mean}} \quad \text{S22}$$

$$\sigma=\sqrt{\frac{1}{n}\sum_{k=1}^{n}(x_k-\bar{x})} \quad \text{S23}$$

where n is the number of measured samples, $x_k$ is the measurement of the sample, and $\bar{x}$ is the mean value of the samples.

# S3 Supplementary Tables

**Table S1.** Geometric parameters

| **Chamber parameters (unit)** | | **Value** |
|---|---|---|
| Inlet inside diameter of the chamber (mm) | | 7.75 |
| Vacuum pump outlet inside diameter (mm) | | 24 |
| Length, height and width of the chamber (mm) | | 350/200/200 |
| Diameter of circular baffle (mm) | | 50 |
| Distance from the chamber inlet to circular baffle (mm) | | 20 |
| Thickness of Baffle A or B or C or P (mm) | | 2 |
| Distance from the chamber inlet to Baffle A/B/C/P (mm) | | 100/175/250/300 |
| **3D object parameters (unit)** | | **Value** |
| Upper section | Diameter (mm) | 25 |
| | Height (mm) | 80 |
| Lower section | Diameter (mm) | 165 |
| | Height (mm) | 20 |

**Table S2.** Lennard–Jones parameters for the gaseous species[4,5]

| Species | Collision diameter $\sigma$ ($10^{-10}$ m) | Potential well depth $\varepsilon/k_B$ (K) | Ref. |
|---|---|---|---|
| Ar | 3.33 | 136.5 | 4 |
| TMA | 5.3 | 471 | 4 |
| $CH_4$ | 3.774 | 143.81 | 5 |

**Table S3.** Operating parameters of the ALD system of this study

| Parameter | Value |
|---|---|
| $H_2O$ cylinder temperature (°C) | 25 |
| TMA cylinder temperature (°C) | 25 |
| Manifold temperature (°C) | 110 |
| Chamber temperature (°C) | 150 |
| Ar mass flow rate (sccm)* | 400 |
| Reactor pressure (Pa) | 170 |
| TMA valve opening time (s) | 0.02 |

*sccm is the abbreviation for cubic centimeters per minute in the standard state. The MFC standard conditions are 0°C and $1.01325 \times 10^5$ Pa.

**Table S4.** Surface chemistry parameters

| Parameter | Value | Ref. |
|---|---|---|
| Reactive hydroxyl concentration [6], $C_{OH,0}$ | $1.25\times10^{-5}$ mol $m^{-2}$ | 6 |
| Sticking coefficient [7], $s_i$ | 0.0064 | 7 |
| Adsorption activation energy of TMA [8] | 0 eV | 8 |
| Desorption activation energy of TMA [8] | 0.61 eV | 8 |
| Reaction activation energy of TMA [8] | 0.52 eV | 8 |

**Table S5.** Kinetic corrosion parameters

| Sample | $E_{corr}$ (V *vs*. RHE） | $I_{corr}$ ($A\ cm^{-2}$) | $\beta_a$ (mV) | $\beta_c$ (mV) | $R_p$ ($k\Omega\ cm^2$) |
|---|---|---|---|---|---|
| No deposition | -0.415 | $4.40\times10^{-5}$ | 1549.3 | 273.85 | 2297.77 |
| Ideal ALD, unetched | 0.487 | $2.23\times10^{-5}$ | 1495.7 | 826.19 | 10356.46 |
| Ideal ALD etch 5min | -0.145 | $6.34\times10^{-5}$ | 3553.4 | 345.75 | 2157.12 |
| Non-ideal ALD, unetched | 0.077 | $2.55\times10^{-5}$ | 1423.6 | 757.13 | 8403.47 |
| Non-ideal ALD, etched 5min | -0.336 | $6.31\times10^{-5}$ | 3528.9 | 618.84 | 3622.58 |

Here, $\beta_a$ and $\beta_c$ are the Tafel slopes for anodic polarization and cathodic polarization in the polarization curve, $I_{corr}$ is the corrosion current density, $E_{corr}$ is the corrosion potential, and $R_p$ is the polarization resistance.

**Table S6.** Electrochemical data obtained via equivalent circuit fitting of the EIS curves

| Sample | $R_S$ ($\Omega$ cm$^2$) | $C_{po}$ (F cm$^{-2}$) | $R_{po}$ ($\Omega$ cm$^2$) | $C_{dl}$ (F cm$^{-2}$) | $R_{ct}$ ($\Omega$ cm$^2$) | Error % |
|---|---|---|---|---|---|---|
| No deposition | 8.03 | $8.93\times10^{-5}$ | 751.9 | $8.06\times10^{-5}$ | 3999 | 0.78 |
| Ideal ALD, unetched | 31.89 | $5.77\times10^{-5}$ | 593 | $6.82\times10^{-5}$ | $1.92\times10^{6}$ | 1.26 |
| Ideal ALD, etched 5min | 8.25 | $9.12\times10^{-5}$ | 235.7 | $4.05\times10^{-6}$ | 6108 | 1.02 |
| Non-ideal ALD, unetched | 30.92 | $6.12\times10^{-5}$ | 838.9 | $7.80\times10^{-5}$ | 20197 | 0.87 |
| Non-ideal ALD, etched 5min | 23.37 | $9.53\times10^{-5}$ | 839.8 | $2.98\times10^{-5}$ | 3577 | 0.18 |

$R_S$ represents the resistance of the solution; $R_{po}$ is the resistance of the film, reflecting the film's ability to block the electrolyte; $C_{po}$ is the capacitance of the film, indicating the film's resistance to permeation. $R_{ct}$ represents the charge transfer resistance, while $C_{dl}$ is the double-layer capacitance at the interface. Both $R_{ct}$ and $C_{dl}$ reflect the electrochemical reactions related to charge transfer and double-layer diffusion.

## S4 Supplementary Figures

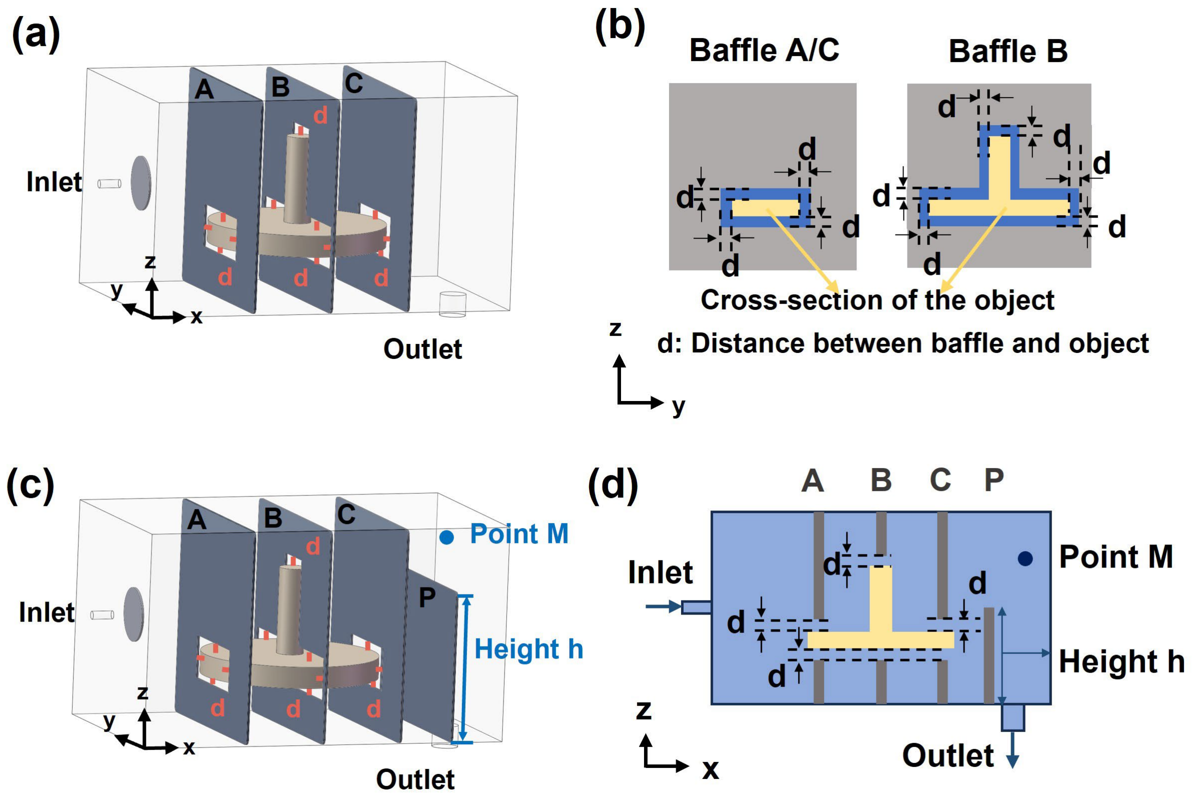


**Figure S1.** Schematic diagram of adding (a) Baffles A, B, C and (c) Baffles A, B, C, P; (b) z-y axis views of Baffles A, B, and C (same with A); (d) Cross-section at y = 100 mm after adding Baffles A, B, C, P (h is the height of Baffle P and point M is 20 mm from the top of the chamber).

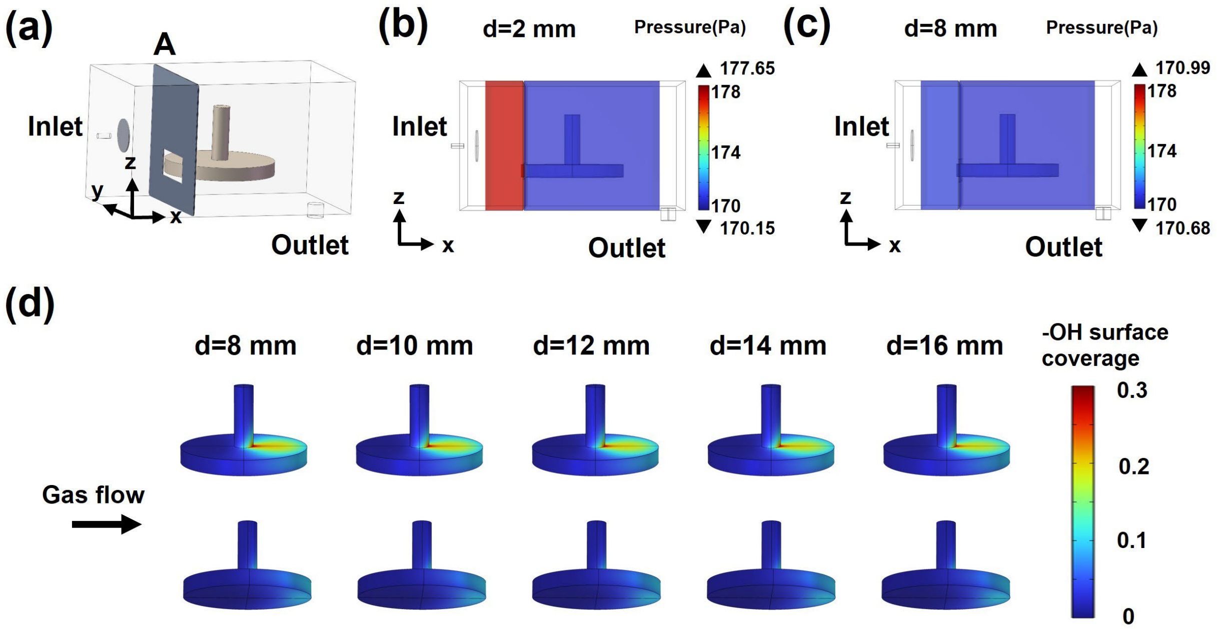


**Figure S2.** (a) Schematic diagram of Baffle A addition; (b) Pressure distribution in the chamber at d = 2 mm; (c) Pressure distribution in the chamber when $\Delta p < 0.5$ Pa first occurs at d = 8 mm; (d) -OH surface coverage at different d.

5

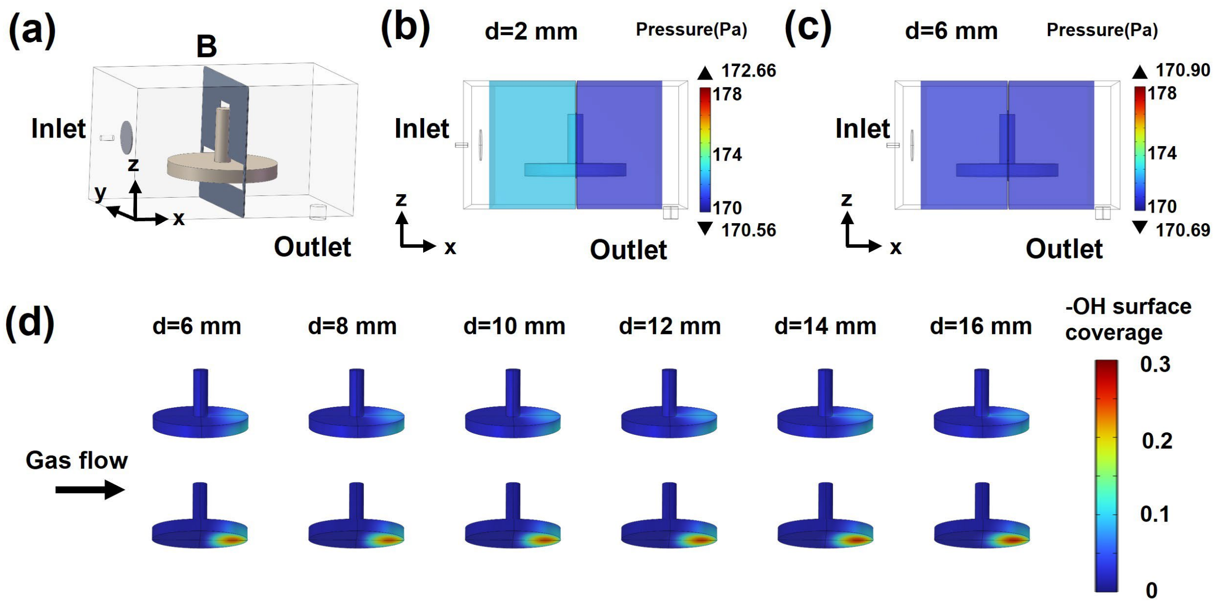


**Figure S3.** (a) Schematic diagram of Baffle B addition; (b) Pressure distribution in the chamber at d = 2 mm; (c) Pressure distribution in the chamber when $\Delta p < 0.5$ Pa first occurs at d = 6 mm; (d) -OH surface coverage at different d.

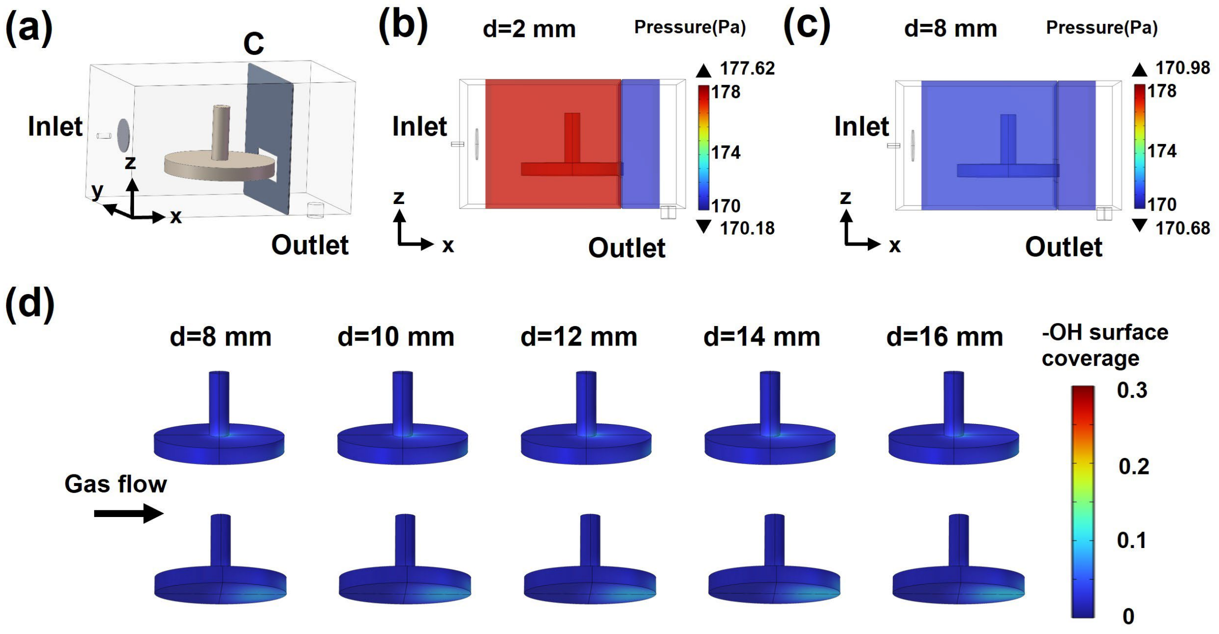


**Figure S4.** (a) Schematic diagram of Baffle C addition; (b) Pressure distribution in the chamber at d = 2 mm; (c) Pressure distribution in the chamber when $\Delta p < 0.5$ Pa first occurs at d = 8 mm; (d) -OH surface coverage at different d.

5

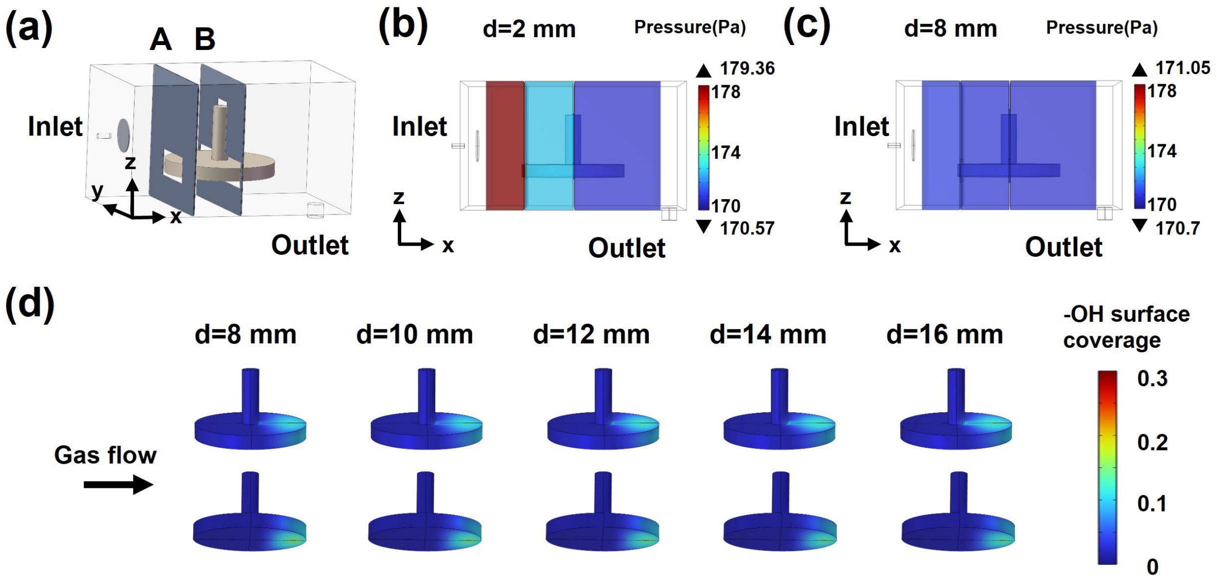


**Figure S5.** (a) Schematic diagram of Baffles A, B addition; (b) Pressure distribution in the chamber at d = 2 mm; (c) Pressure distribution in the chamber when $\Delta p < 0.5$ Pa first occurs at d = 8 mm; (d) -OH surface coverage at different d.

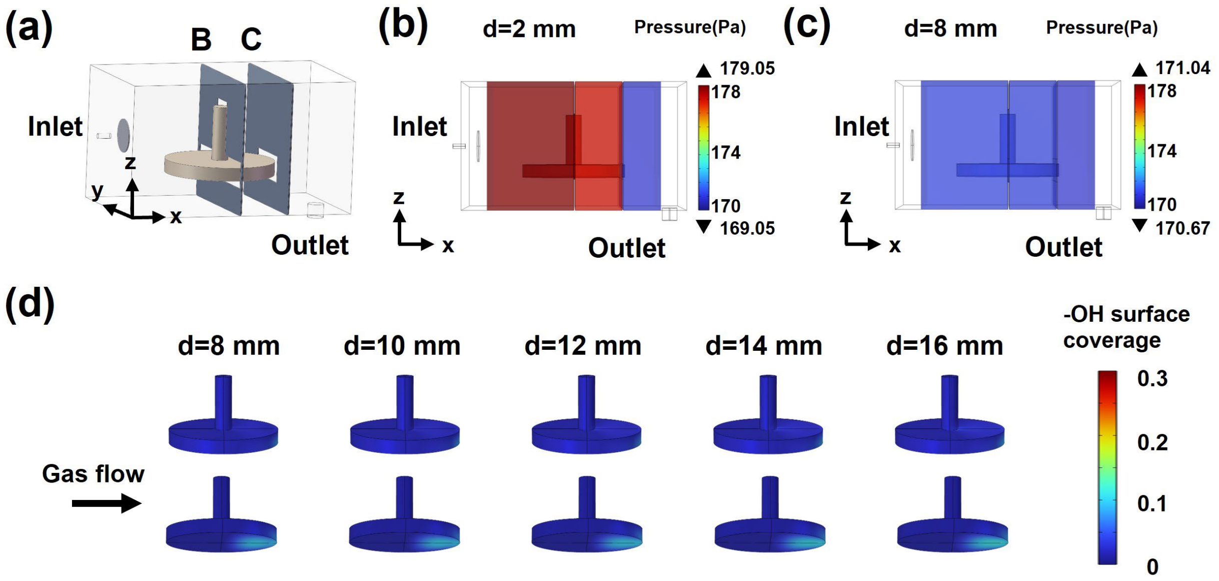


**Figure S6.** (a) Schematic diagram of Baffles B, C addition; (b) Pressure distribution in the chamber at d = 2 mm; (c) Pressure distribution in the chamber when $\Delta p < 0.5$ Pa first occurs at d = 8 mm; (d) -OH surface coverage at different d.

5

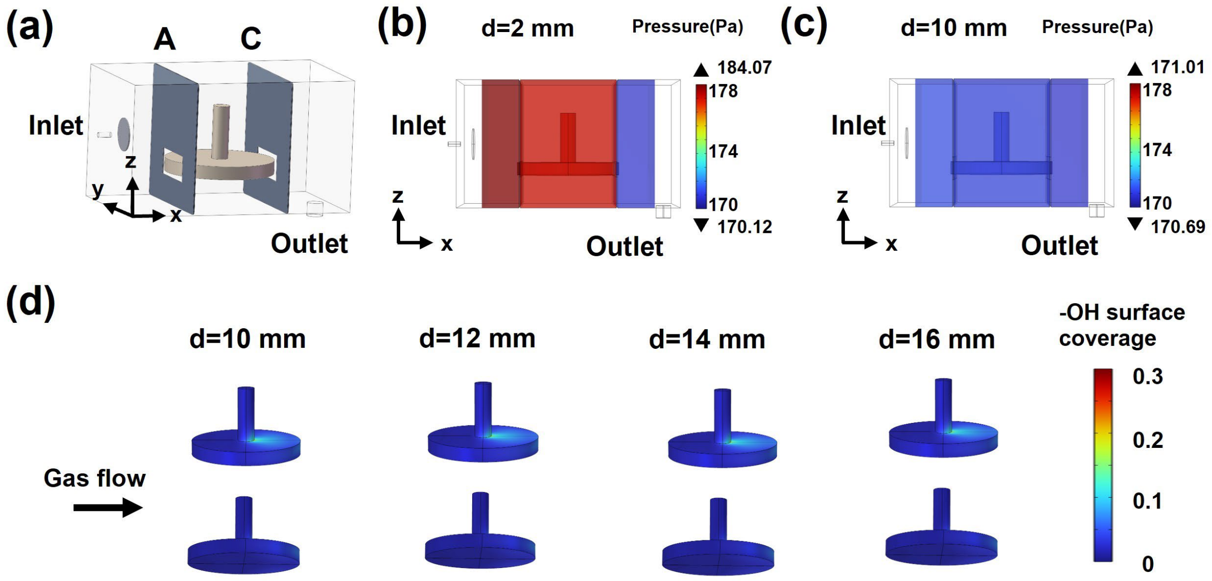


**Figure S7.** (a) Schematic diagram of Baffles A, C addition; (b) Pressure distribution in the chamber at d = 2 mm; (c) Pressure distribution in the chamber when $\Delta p < 0.5$ Pa first occurs at d = 10 mm; (d) -OH surface coverage at different d.

5

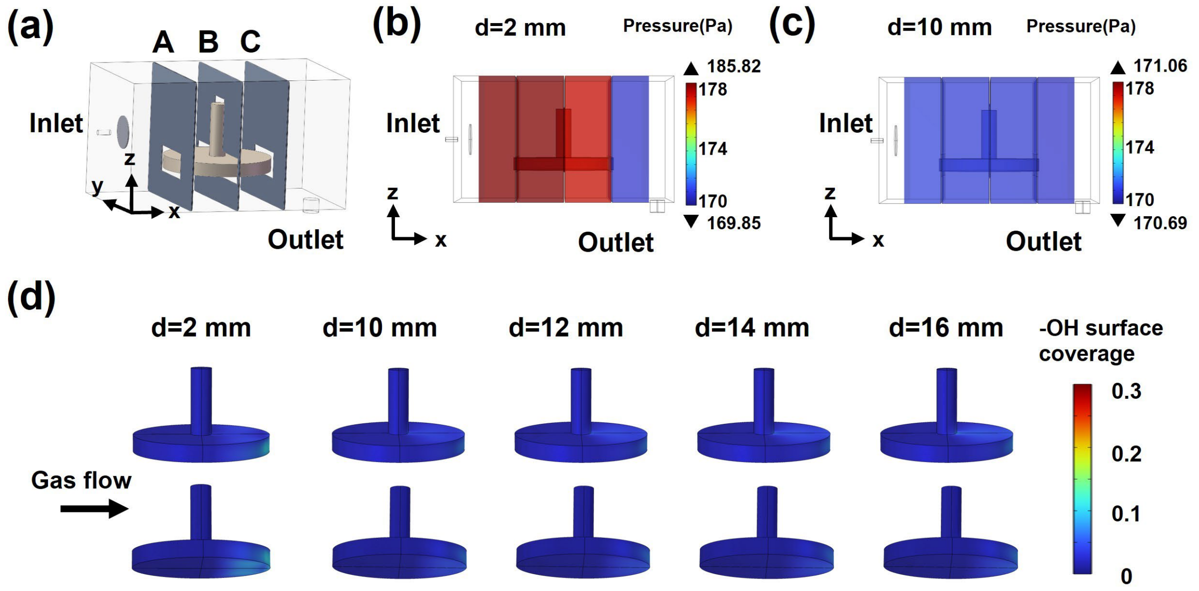


**Figure S8.** (a) Schematic diagram of Baffles A, B, C addition; (b) Pressure distribution in the chamber at d = 2 mm; (c) Pressure distribution in the chamber when $\Delta p < 0.5$ Pa first occurs at d = 10 mm; (d) -OH surface coverage at different d.

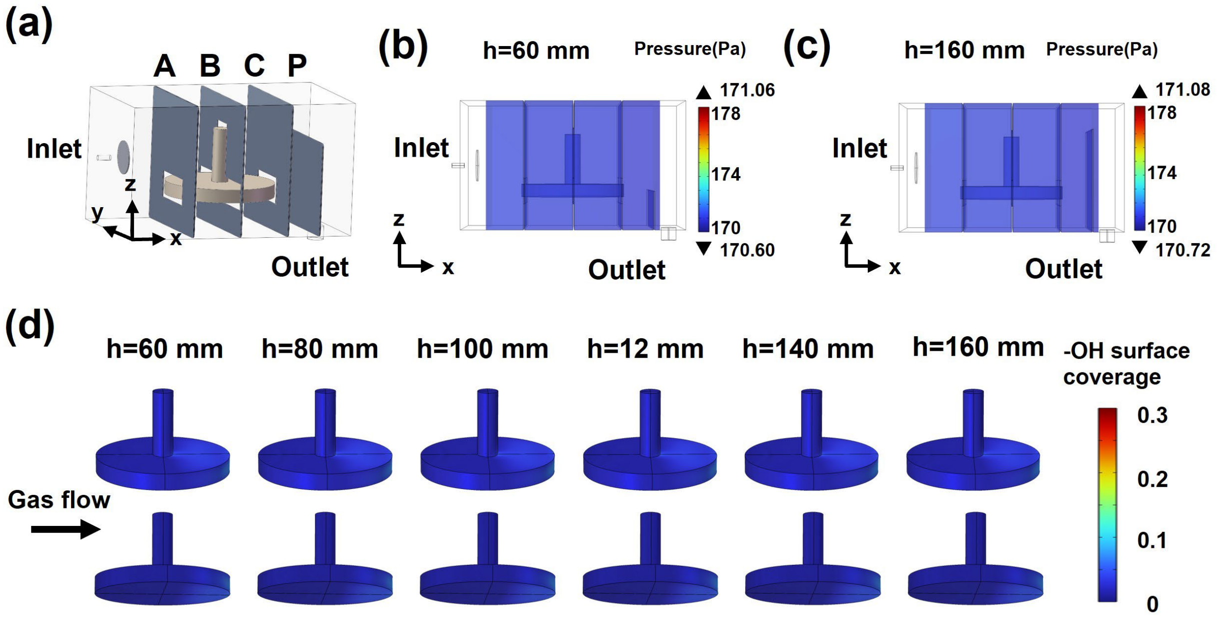


**Figure S9.** (a) Schematic diagram of Baffles A, B, C, P addition; (b) Pressure distribution in the chamber at h = 60 mm; (c) Pressure distribution in the chamber at h = 160 mm; (d) -OH surface coverage at different height of baffle P.

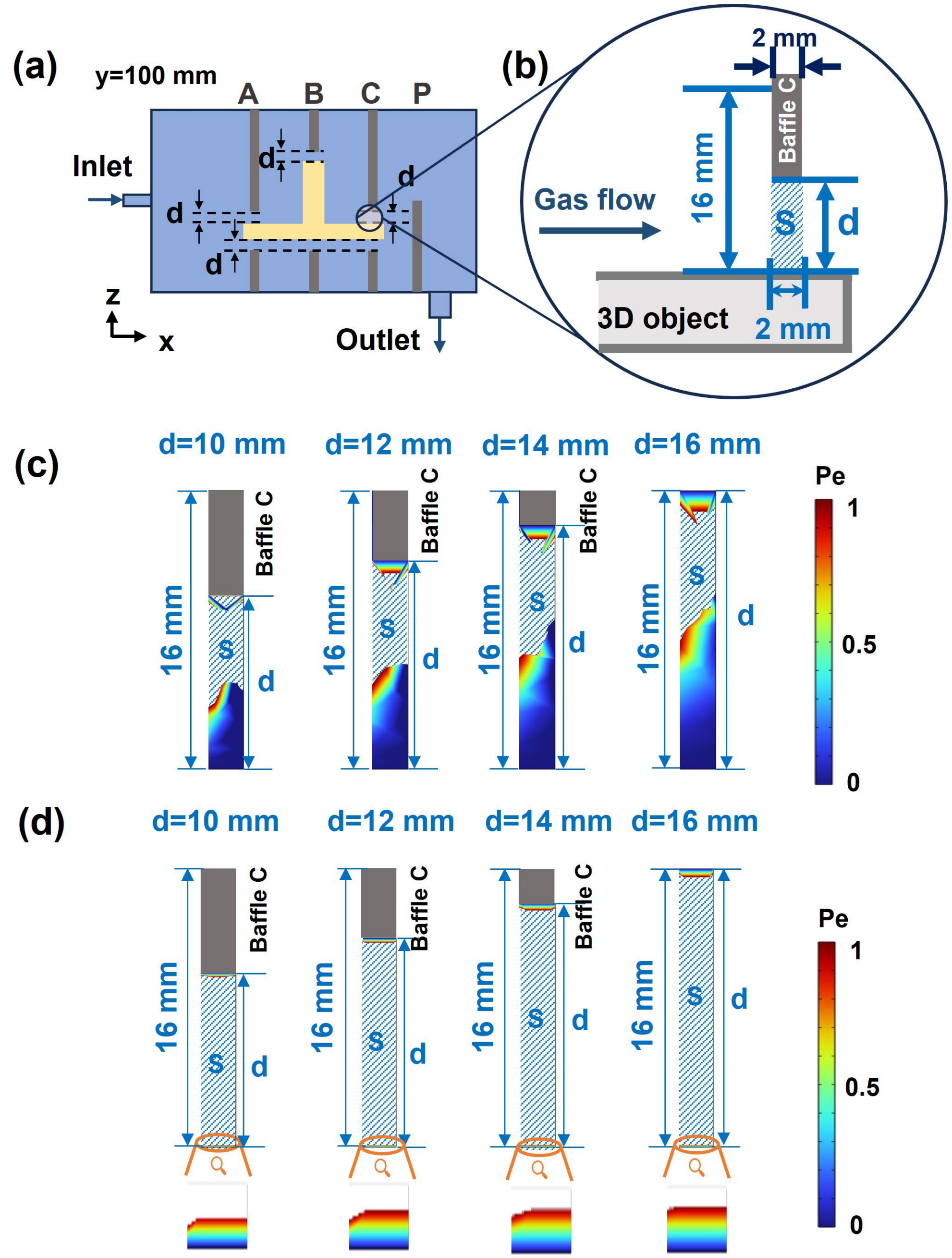


**Figure S10.** (a) Section of chamber with Baffles A, B, C, P at y = 100 mm; (b) Localized magnification of the slit between Baffle C and the complex object (blue shaded area is defined as Region S); Distribution of $Pe < 1$ in the (c) z-axis direction and (d) x-axis direction in the Region S at t = 2.5 s for different d.

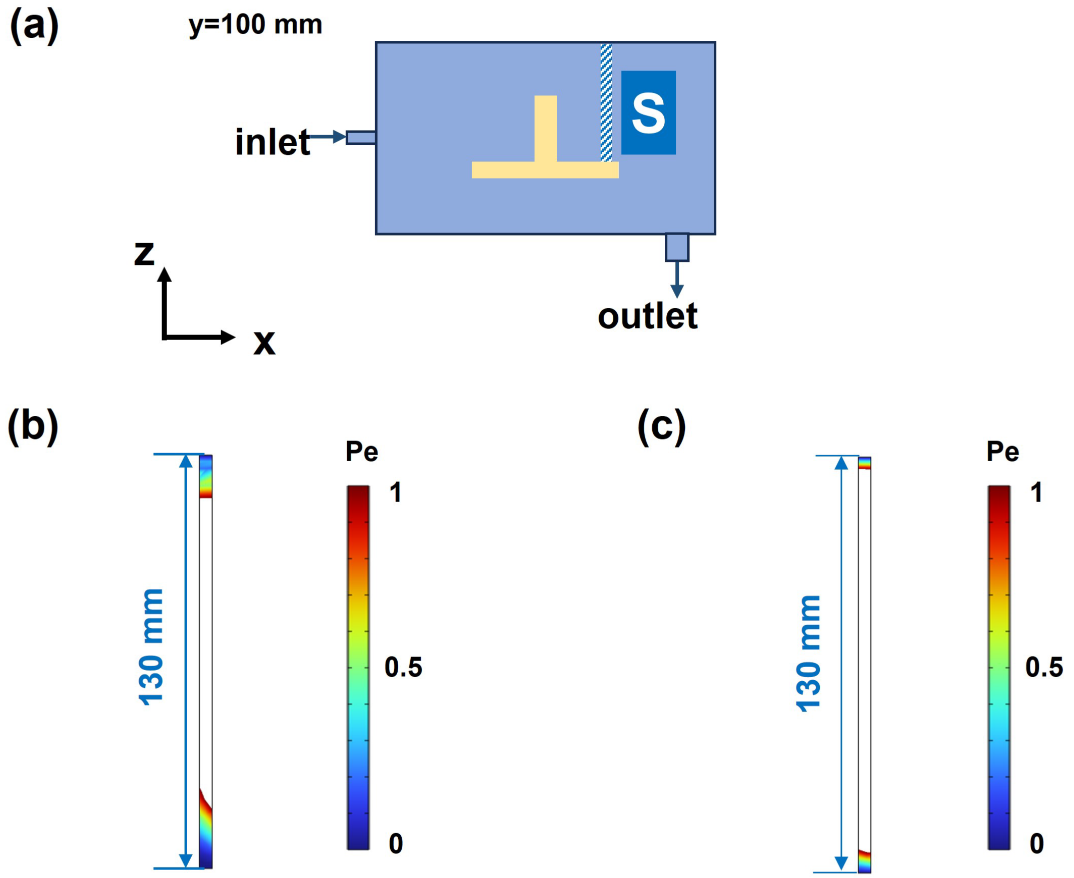


**Figure S11.** (a) Section of chamber with no baffle at y = 100 mm (blue shaded area is defined as Region S); Distribution of *Pe* < 1 in the z-axis direction (c) and x-axis direction (d) in the Region S at t = 2.5 s for different d.

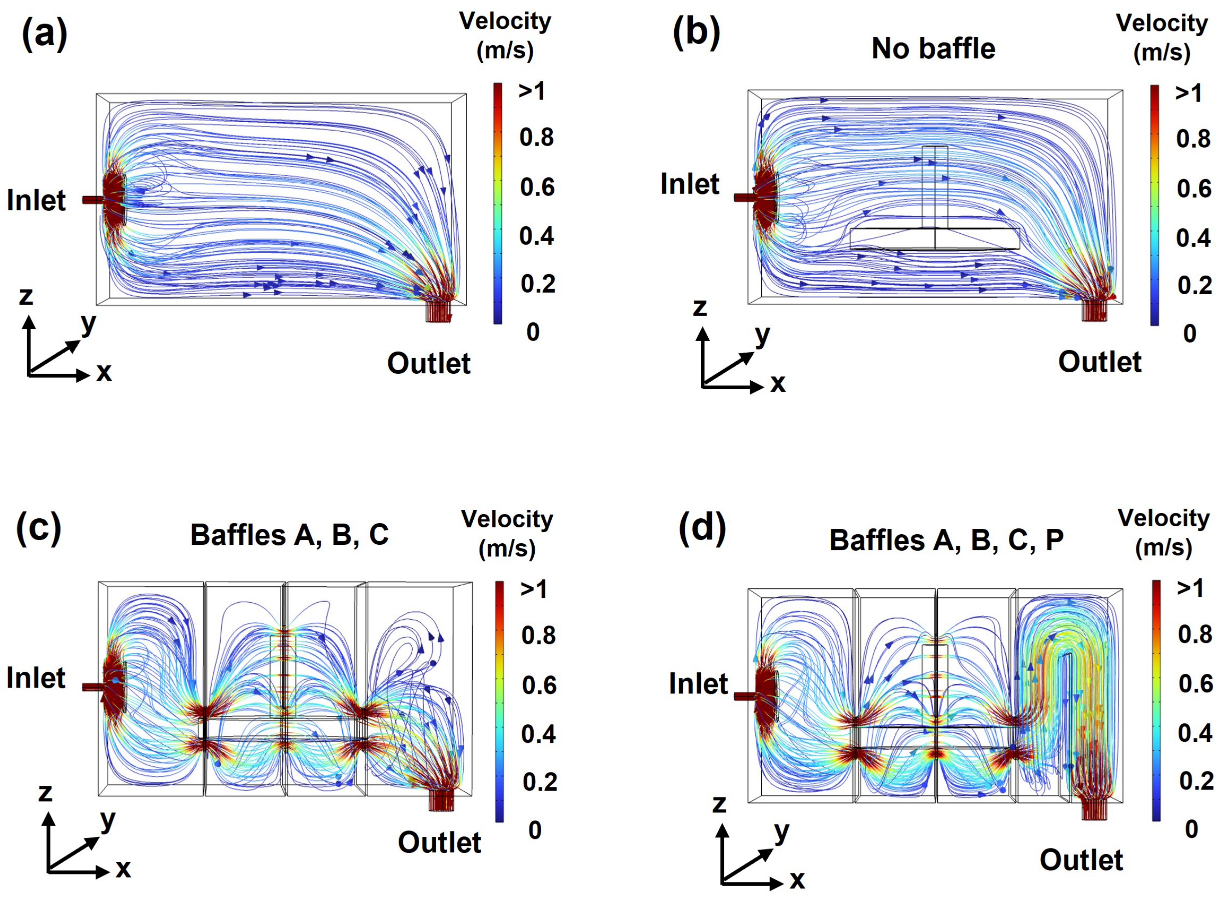


**Figure S12.** Flow field in the chamber (a) without complex object, (b) with complex object, (c) complex object added Baffles A, B, C and (d) complex object added Baffles A, B, C, P.

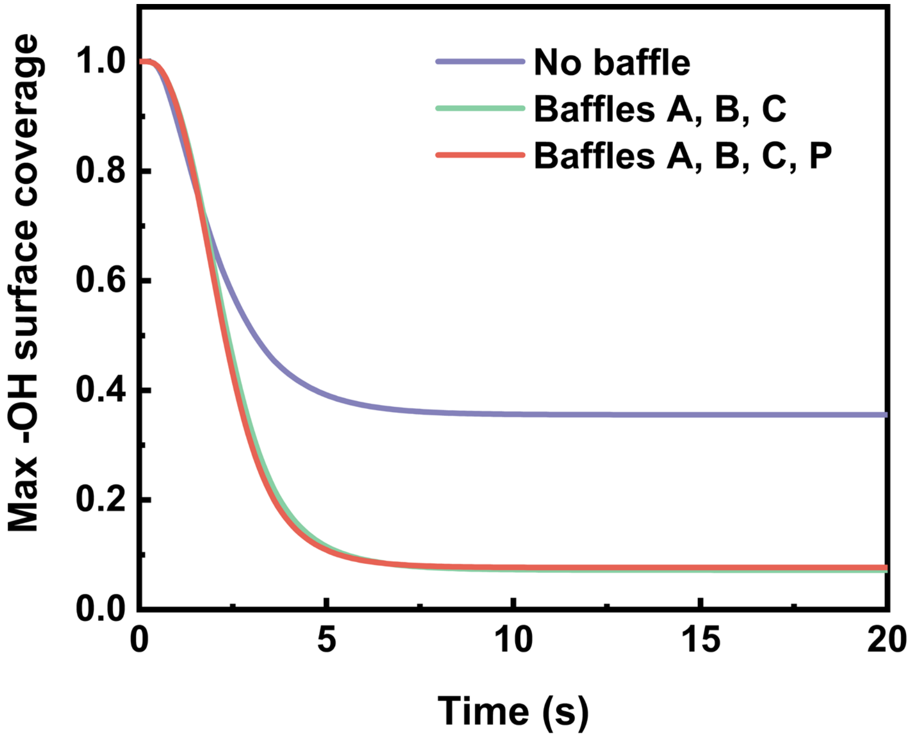


**Figure S13.** Maximum OH surface coverage of complex object in no baffle (purple line), with Baffles A, B, C (green line) and with Baffles A, B, C, P (red line).

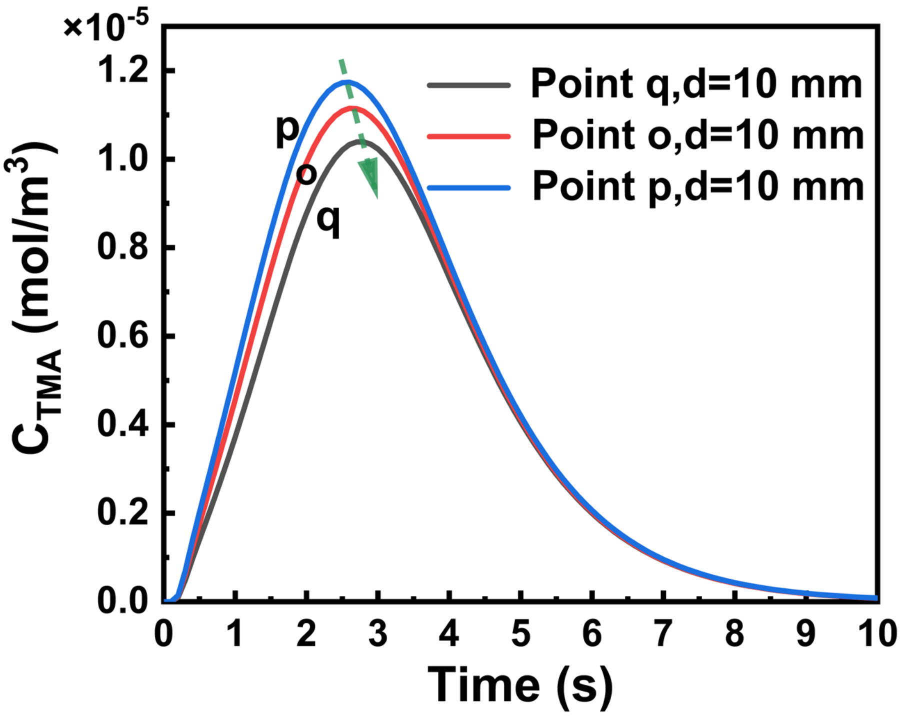


**Figure S14.** TMA concentration at p, o, and q (detail locations are shown in Fig. 1e in the main text).

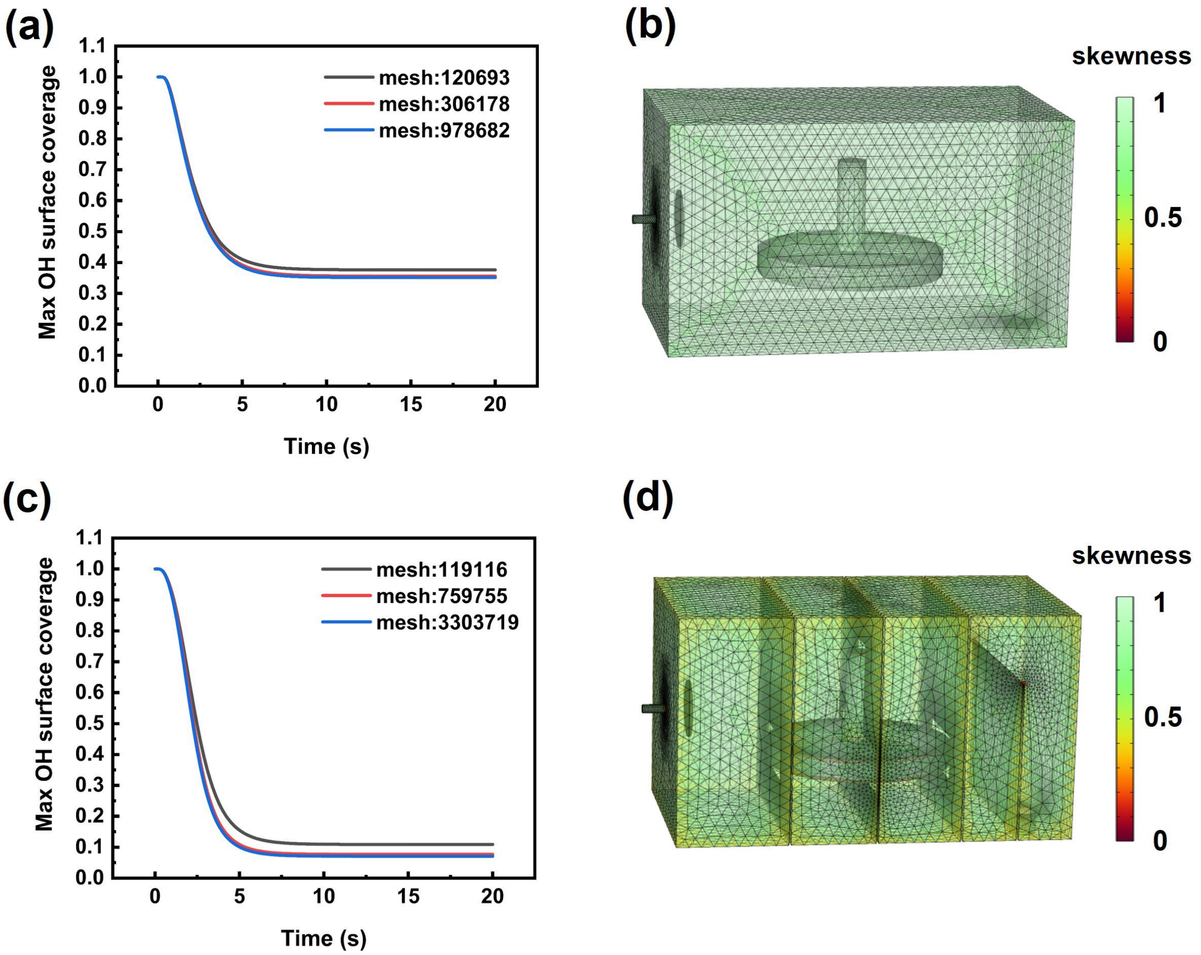


**Figure S15.** Computational mesh grid-independent validation results: (a) without baffles and (c) with baffles; Mesh quality for the reactor: (b) without baffles and (d) with baffles (the closer to 1, the better the mesh quality).

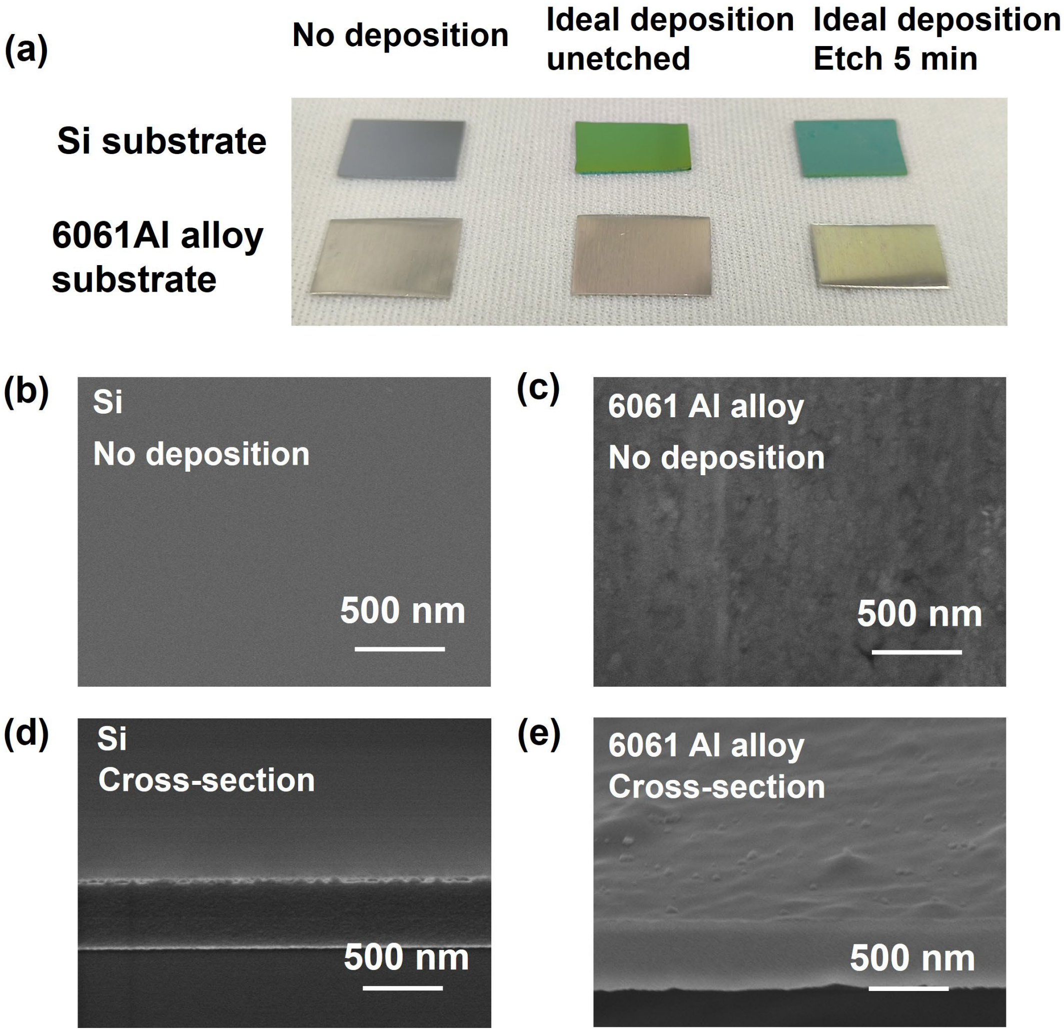


**Figure S16.** (a)Surface morphology on silicon substrate, 6061 aluminum alloy substrate without deposition, 340 nm $Al_2O_3$ deposited, and plasma etching after deposition for 5 minutes; SEM image of (b) Si substrate, (c) 6061 aluminum alloy substrate and cross-section of films on (d) Si substrate, (e) 6061 aluminum alloy substrate after plasma etching.

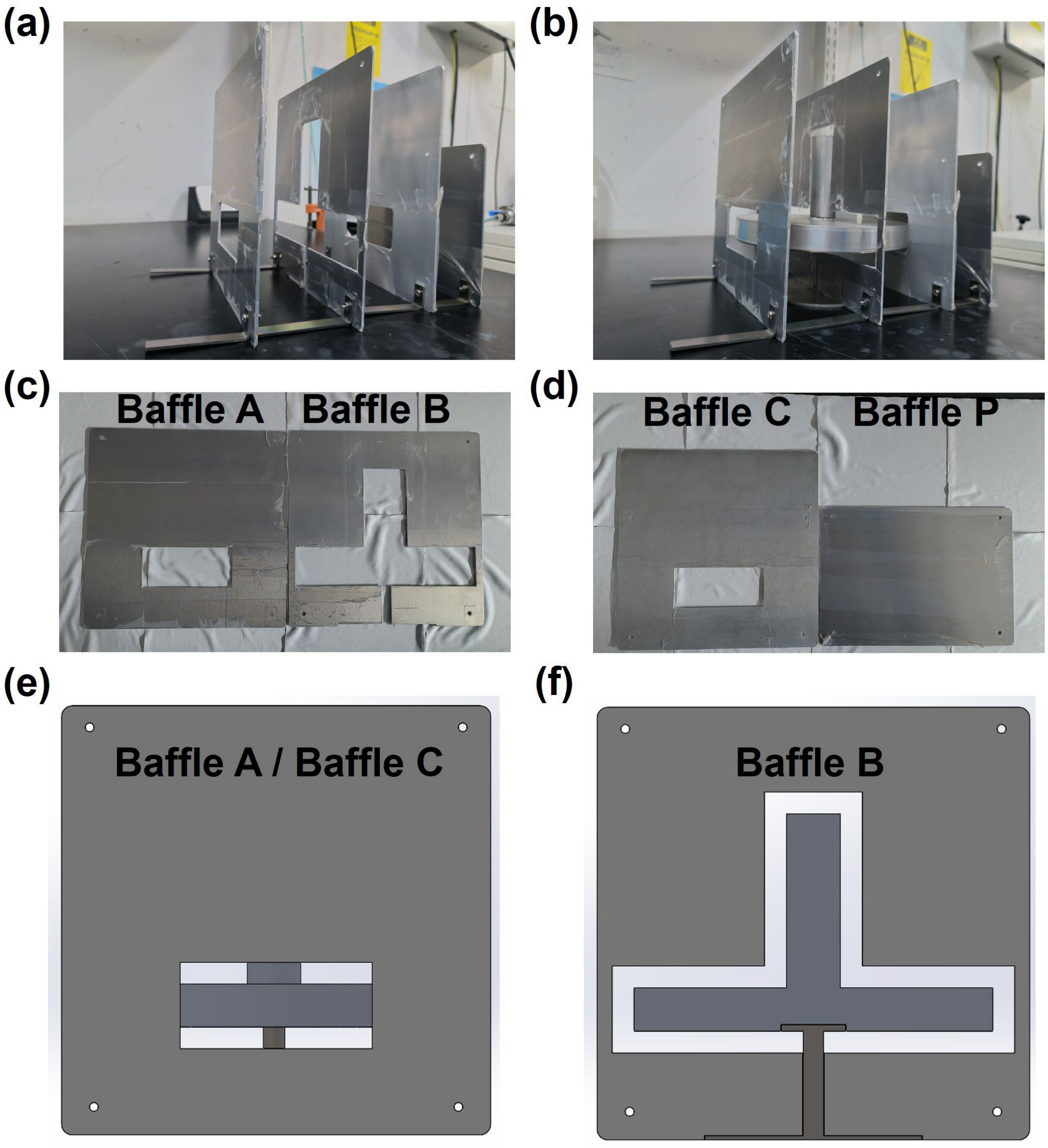


**Figure S17.** Schematic of baffle (surface wrapped with polytetrafluoroethylene (PTFE) tape): (a) Baffles A, B, C, P; (b) Assembly of Baffles A, B, C, P with complex objects; (c) Baffle A and Baffle B; (d) Baffle C and Baffle P; Side view (design schematic) of (e) Baffle A / Baffle C and (f) Baffle B after installation.

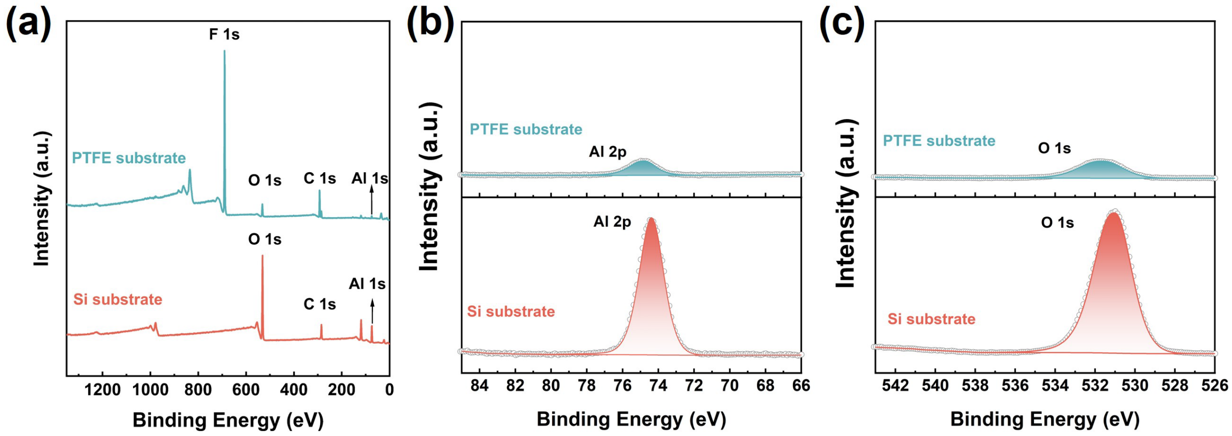


**Figure S18.** Schematic of XPS with different substrate: (a)XPS survey spectra; XPS intensity of (b) Al; (c) O.

5

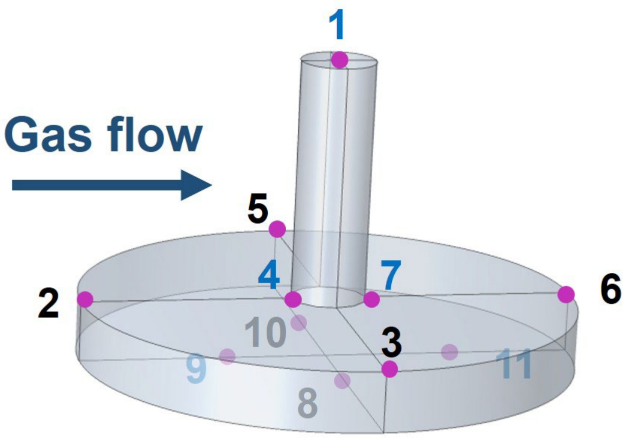


**Figure S19**. Schematic of the 11 sampling positions in the experiment.

Position 1 is located at the center of the top surface;

Position 2, 3, 4, 5, 6 and 7 are located on the upper surface. Position 2, 4, 6 and 7 are collinear and aligned with the direction of fluid flow, while Position 3 and 5 are collinear and oriented perpendicular to the direction of fluid flow;

Position 8, 9, 10, and 11 are situated on the bottom surface, corresponding to the quarter points of the bottom surface's diameter. Position 9 and 11 are collinear and aligned with the direction of fluid flow, while Position 8 and 10 are collinear and oriented perpendicular to the direction of fluid flow.

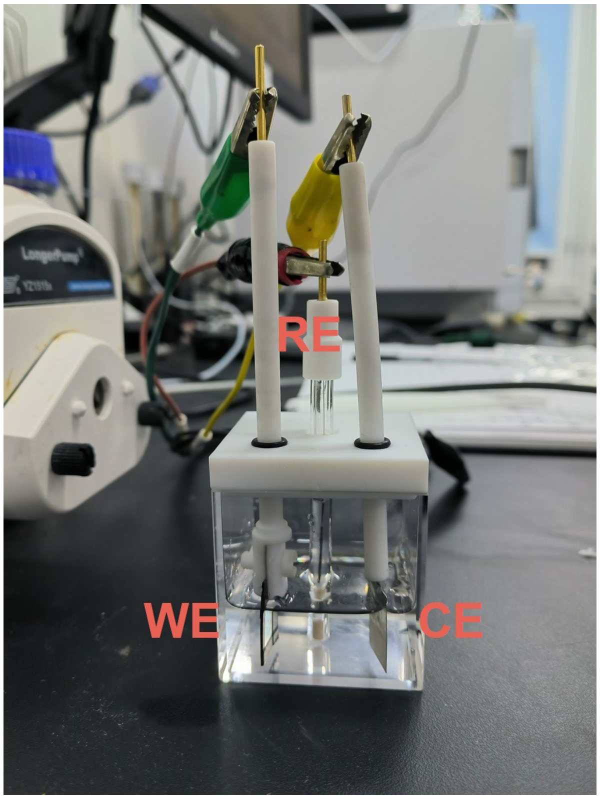


**Figure S20.** Schematic of electrochemical test setup.

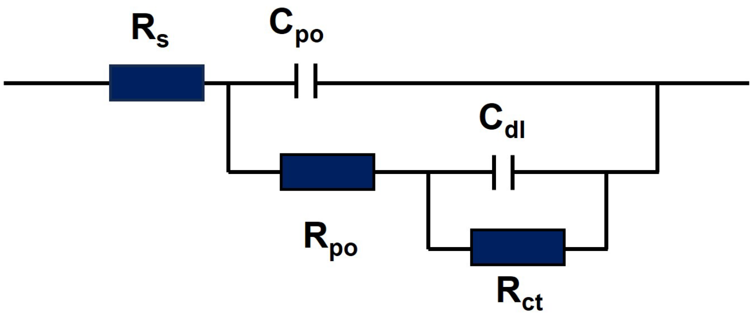


**Figure S21.** Equivalent electric circuits used to fit the EIS measurements at OCP.

$R_S$ represents the resistance of the solution; $R_{po}$ is the resistance of the film, reflecting the film's ability to block the electrolyte; $C_{po}$ is the capacitance of the film, indicating the film's resistance to permeation. $R_{ct}$ represents the charge transfer resistance, while $C_{dl}$ is the double-layer capacitance at the interface. Both $R_{ct}$ and $C_{dl}$ reflect the electrochemical reactions related to charge transfer and double-layer diffusion.

# References


1. Oviroh PO, Akbarzadeh R, Pan D *et al.* New development of atomic layer deposition: Processes, methods and applications. *Sci Technol Adv Mater* 2019; **20**: 465–96.
2. Bird RB, Stewart WE, Lightfoot EN. *Transport Phenomena*. New York: Wiley, 2007.
3. Gakis GP, Vergnes H, Scheid E *et al.* Detailed investigation of the surface mechanisms and their interplay with transport phenomena in alumina atomic layer deposition from TMA and water. *Chem Eng Sci* 2019; **195**: 399–412.
4. Nwanna EC, Coetzee RAM, Jen T-C. A numerical approach on the selection of the purge flow rate in an atomic layer deposition (ALD) process. *Phys Fluids* 2022; **34**: 052003.
5. Tee LS, Gotoh S, Stewart WE. Molecular parameters for normal fluids. Lennard-jones 12-6 potential. *Ind Eng Chem Fund* 1966; **5**: 356–63.
6. Haukka S and Root A. The reaction of hexamethyldisilazane and subsequent oxidation of trimethylsilyl groups on silica studied by solid-state NMR and FTIR. *J Phys Chem* 1994; **98**: 1695–703.
7. Paulson NH, Yanguas-Gil A, Abuomar OY *et al.* Intelligent agents for the optimization of atomic layer deposition. *ACS Appl Mater Interfaces* 2021; **13**: 17022–33.
8. Widjaja Y and Musgrave CB. Quantum chemical study of the mechanism of aluminum oxide atomic layer deposition. *Appl Phys Lett* 2002; **80**: 3304–6.
